# Mechanistic insights into Orai dynamics during pore opening

**DOI:** 10.1080/19336950.2026.2624276

**Published:** 2026-02-08

**Authors:** Hadil Najjar, Veronika Aichner, Magdalena Prantl, Nora Müller, Heinrich Krobath, Isabella Derler

**Affiliations:** a Institute of Biophysics, JKU Life Science Center, Johannes Kepler University Linz, Linz, Austria; b Institute of Theoretical Physics, Johannes Kepler University Linz, Linz, Austria

**Keywords:** Ion channels, SOCE, calcium (Ca^2+^), CRAC channels, STIM1, Orai1, protein dynamics, inter- and intra-transmembrane domain contact sites, hydration, genetic code expansion, crosslinking

## Abstract

Orai channels form highly Ca^2+^-selective pores in the plasma membrane (PM) and represent one of the two essential components of the Ca^2+^ release-activated Ca^2+^ (CRAC) channel. The second component is the Stromal Interaction Molecule (STIM) proteins, which is located in the endoplasmic reticulum (ER). Ca^2+^ influx through CRAC channels serves as the primary route of Ca^2+^ entry into the cell, playing a critical role in downstream signaling pathways such as gene transcription and cell proliferation. Activation of Orai channels is tightly coupled to the depletion of ER Ca^2+^ stores, which triggers STIM proteins to oligomerize and adopt an extended conformation that spans the ER–PM junction, enabling direct interaction with and activation of Orai. Several studies have shown that Orai activation is mediated by global conformational changes across the entire channel complex. In recent years, detailed functional analyses, structural investigations, genetic code expansion techniques, and molecular dynamics simulations have further refined our understanding of the molecular mechanisms underlying Orai1 pore opening and the associated amino acid-level conformational dynamics. In this review, we highlight proposed mechanisms, dynamic features, and functionally relevant contact sites across the Orai1 channel complex that contribute to gating and ion permeation, while also summarizing outstanding questions that remain to be resolved.

## Introduction

Calcium (Ca^2+^) is an essential regulator of numerous cellular and physiological processes, including transcription, immunity, secretion, muscle contraction, and neuronal signaling [1–8]. To function as a second messenger, Ca^2+^ relies on a coordinated network of transporters, ion channels, and Ca^2+^-binding proteins. These proteins actively maintain low cytosolic Ca^2+^ concentrations under resting conditions and enable spatiotemporally controlled elevations in response to signaling cues [4,6,9–12]. Disruption of this Ca^2+^ homeostasis can lead to pathogenic deviations of intracellular Ca^2+^ levels, contributing to diseases such as immune deficiencies and cancer [5,13–18]. Transient increases in cytosolic Ca^2+^ concentration often occur in response to depletion of intracellular Ca^2+^ stores, particularly those of the endoplasmic reticulum (ER) or due to Ca^2+^ influx from the extracellular environment. The Ca^2+^ release-activated Ca^2+^ (CRAC) channel activation serves as the main contributor to the more general store-operated Ca^2+^ entry (SOCE) pathway, which is solely triggered by ER Ca^2+^ store depletion [14]. The pathway serves as a critical mechanism for supplying Ca^2+^ to support downstream signaling processes, including gene expression, proliferation, and cytokine release [14]. Additionally, SOCE plays a vital role in replenishing ER Ca^2+^ stores, thereby restoring the resting state of the cell [19–21]. The latter process involves also other key players, such as plasma membrane Ca^2+^ ATPases (PMCA) [22,23] and sarco-/endoplasmic reticulum Ca^2+^ ATPases (SERCA) [9,24].

## The CRAC channel composition

The CRAC channel consists of two essential components: the Ca^2+^ sensor Stromal Interaction Molecule (STIM), located in the ER membrane [25,26], and the pore-forming subunit Orai, located in the plasma membrane (PM) [3,5,14,27–29]. Two STIM homologs (STIM1, STIM2) and three Orai homologs (Orai1, Orai2, Orai3) have been identified [26,30]. Increasing evidence suggests that CRAC channels assemble as physiological heteromers [31–33], with heteromeric channel complex composition fine-tuning the amplitude, duration, and shape of Ca^2+^ signals [6,32–37].

The different STIM and Orai isoforms are ubiquitously expressed, while their relative abundance varies significantly across tissues depending on cell type and differentiation state [38–47]. STIM proteins function as ER Ca^2+^ sensors, detecting decreases in luminal Ca^2+^ concentration [3,14], which trigger their conformational rearrangement and oligomerization in the cytosolic STIM domains [48–52]. Activated STIM is initially targeted via PIP_2_ to the PM, finally allowing its direct interaction with Orai channels [5] and facilitating their translocation and accumulation at ER-PM junctions [48,51,53–56]. Upon recruitment, Orai channels undergo global conformational changes [57,58], and open their Ca^2+^-selective pore allowing Ca^2+^ influx into the cytosol [5,14,26,34,53,59–64].

Tissue-specificity and the specialized cellular roles of physiological CRAC channels are shaped not only by the repertoire of STIM and Orai isoforms, but also by accessory proteins [14,65–69], post-translational modifications [70–81] and alterations in lipid metabolism [69,82–85]. For instance, local Ca^2+^ entry via Orai1 that drives activation of the transcription factor of nuclear factor of activated T cells (NFAT) is orchestrated by the scaffolding protein AKAP79 (A-kinase anchoring protein 79). AKAP79 is directly bound to Orai1 N-terminus and recruits signaling components, positioning NFAT1 near Orai1 to enable efficient, microdomain-restricted NFAT signaling [86]. A detailed discussion of the modulatory factors is beyond the scope of this review and has been covered elsewhere [3,65,77,87–89]. The physiological importance of this system is underscored by the fact that both gain-of-function (GoF) and loss-of-function (LoF) mutations in the CRAC channel components can lead to severe pathologies, including Stormorken-like syndrome (OMIM #185070), York syndrome [90], tubular aggregate myopathy (TAM; OMIM # 615,883), or severe combined immunodeficiency (SCID; OMIM # 610,277) [11,28,91–94].

A detailed understanding of the molecular mechanisms underlying CRAC channel activation is therefore essential for elucidating the pathogenesis of associated disorders and for developing targeted therapeutic strategies, particularly in conditions such as acute pancreatitis [95–97], acute brain injury [98] or cancer [99]. While STIM1 and Orai1 are the most extensively studied components of the CRAC channel, several aspects of their molecular mechanisms remain incompletely understood. This review highlights recent advances in uncovering the regulatory principles governing Orai1 channel gating and outlines critical questions that must be addressed to achieve a complete understanding of its regulation and physiological roles.

### STIM

STIM proteins are single-pass transmembrane (TM) proteins localized to the ER membrane [25,53]. Two homologs, STIM1 and STIM2, have been identified, along with several splice variants [41,42,100–104]. The ER luminal domain of STIM comprises two EF-hand motifs and a sterile alpha motif (SAM) domain [25,53,105], both of which are essential for Ca^2+^ sensing and homomerization [49,102,106–108]. A short TM region of STIM1 spans the ER membrane, linking the luminal N-terminus with the cytosolic C-terminus [25,26,102]. The cytosolic region features multiple coiled-coil (CC) domains and C-terminal segments that facilitate protein–protein and protein–lipid interactions [60,61,109–115]. Specifically, three CC domains (CC1-CC3) have been identified, with CC1 further subdivided into CC1α1–α3 [102,116,117]. Multiple segments within these three CC domains of STIM1 have been characterized, revealing regions crucial for maintaining the quiescent state and domains required for STIM1-Orai1 coupling. Fragments including the CRAC activation domain (CAD, STIM1_342–448_) [61], the Orai-activating small fragment (OASF, STIM1_233–474_) [52], the STIM-Orai-activating region (SOAR, STIM1_344–442_) [60], and the CC domain containing region b9 (Ccb9, STIM1_339–444_) [118] represent the minimal cytosolic segments with the inherent ability to couple to and activate Orai channels [52,54,60,61,119]. A flexible linker between CC2 and CC3, referred to as the apex [120,121], has been shown to play a critical role in Orai activation [5,14,102,122,123].

In the resting state, STIM1 adopts a compact, domain-swapped trans-dimeric conformation, stabilized by several autoinhibitory mechanisms [48,51,119,124–129]. These include Ca^2+^-bound EF-hand motifs [130], electrostatic interactions between the CAD domain and the ER membrane [131], and an auto-inhibitory clamp formed through intermolecular contacts between the CC1α1 and CAD domains [48,51,132–134]. Additionally, hydrophobic and electrostatic interactions between the two CC1α1/3 regions further reinforce the inactive conformation [48,119,125,126,135,136]. Under resting conditions, the cytosolic C-terminal region of STIM1 remains folded near the ER membrane [131,137]. Upon ER Ca^2+^ store depletion, Ca^2+^ dissociates from the EF-hand motifs, initiating a cascade of conformational rearrangements [48–51] transmitted through the TM domains to the cytosolic C-termini [94,105,130,132,138–140]. This includes close dimerization of CC1α1 domains, which disrupts the inhibitory clamp and exposes the CAD/SOAR domain [48,128,135]. The exposed CAD/SOAR promotes further homomerization [48,51,52,126,141], translocation of STIM to ER–PM junctions, and binding to Orai1 channels [5], thereby enabling CRAC channel activation [102,119,122,123,128,129,132,136].

Although a full-length structure of STIM1 is not yet available, structural data for several individual domains and fragments exist, including NMR structures of Ca^2+^-bound STIM1 and STIM2 N-terminal segments [49,105], three X-ray structures (human CC1 [127], human SOAR [117] and the full CC region of *C. elegans* STIM1 [117]) as well as two NMR structures (human CC1α1–3 [128] and CC1α3-CC2 [141]), which are proposed to represent distinct conformational states: resting, active, or intermediate. Moreover, various research groups have integrated these fragments into computational models to provide a comprehensive structural framework that approximates the full-length protein and elucidates its activation mechanism [83,94,129,131,137,138,140,142,143].

### Orai

**Figure 1.**
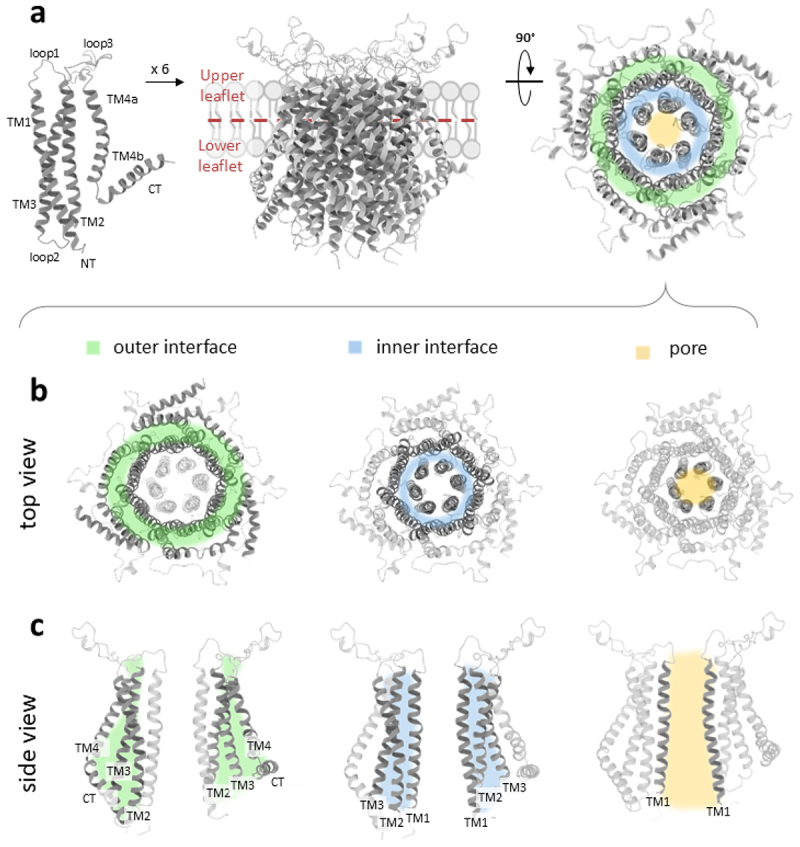
Structural overviews of the Orai1 channel homology model based on the X-ray dOrai structure highlighting the outer interface, inner interface, and the pore.

Orai functions as the pore-forming subunit of the CRAC channel [28,30,144–147]. To date, four crystal structures and two cryo-electron microscopy (cryo-EM) structures have been resolved for the *Drosophila melanogaster* Orai (dOrai) channel [148–151], which share a high degree of homology with human Orai1 (~73% sequence identity), with the highest conservation found within the four TM domains. All resolved structures consistently reveal a supramolecular assembly with hexameric symmetry of the dOrai channel, featuring a central pore-forming region at the core of the complex. Among the closed-state structures, two represent wild-type dOrai (PDB: 4HKR) [149], while one corresponds to a LoF mutant, dOrai K163W (PDB: 6BBG, 6BBH, 6BBI) [150], the analogue of human Orai1 R91W [149]. Based on the crystal structure of dOrai (PDB: 4HKR) [149], a homology model of human Orai1 (Figure 1(a)), representing its closed state, was generated [152]. The open-state structures were obtained via single-point mutations, specifically H206A (X-ray; PDB: 6BBF [150], cryo-EM; PDB: 7HR5 [151]) and P288L (cryo-EM; PDB: 6AKI) [148], which correspond to H134A and P245L in human Orai1, respectively. Although these structures have significantly advanced our understanding of inter- and intramolecular interactions within Orai and between Orai and STIM, high-resolution structures of human Orai variants remain a critical unmet need. In this review, we refer to dOrai exclusively in the context of structural data, while functional analyses are restricted to human Orai channels. For clarity and consistency, we denote the human variant simply as Orai1.

a) Cartoon representing a single Orai1 subunit with the TM regions (TM1, TM2, TM3, TM4a and TM4b), the cytosolic regions (NT, loop2 and CT), and extracellular regions (loop1 and loop3) (left). Schematic representations of the side (middle) and top (right) views of the hexameric Orai1 assembly (based on PDB: 4HKR) in the lipid bilayer. The top view highlights three key interfaces: the outer interface between TM2/3 and TM4 (green-shaded region), the inner interface between TM2/3 and TM1 (blue-shaded region), and the pore formed by TM1 (yellow-shaded region). b–c) Individual visualization of the three interfaces is presented via top views of the Orai1 hexamer (b) and a cross-sectional view of the hexameric Orai1 channel displaying two opposing subunits (c). TM domains contributing to each respective interface are shown in dark gray, while nonparticipating TM domains are depicted in light gray. The same color code as in (a) is used for the respective interfaces.

Each Orai1 subunit comprises four TM domains connected by three loops: two extracellular loops (linking TM1 to TM2 (loop1) and TM3 to TM4 (loop3)) and one intracellular loop (connecting TM2 to TM3 (loop2)) (Figure 1(a), left). Both the N-terminus and C-terminus of the subunit reside in the cytosol (Figure 1(a), left). The TM helices of the hexameric Orai1 complex are arranged concentrically (Figure 1, middle, right). At the core of the channel complex, six TM1 helices form the central ion-conducting pore [149](Figure 1(a-c), right). The TM1 extends into the cytosol as a helical segment [28], referred to as the extended transmembrane Orai1 N-terminal (ETON) region [153]. Surrounding this central ring is a second layer composed of tightly packed TM2 and TM3 helices [64,149], which facilitate communication between the pore and the outermost ring of six TM4 helices [64,154]. Each TM4 helix is subdivided into two segments, TM4a and TM4b (Figure 1, a left), separated by a kink at residue P245. The TM4b segment includes a cytosolic hinge region [149], known as the nexus (^261^LVSHK^265^) [155], which is highly conserved and connects TM4 to its extended C-terminal region (TM4ext), also called C-terminus [148–151,156](Figure 1(a), left). In addition to the transmembrane (TM) domains forming interfaces with one another, specifically TM1 with TM2 and TM3 (TM1-TM2/3) and TM2/3 with TM4, the nexus region also forms an interface with TM3 close to the cytosol. This interface can be functionally divided into two distinct regions: the lower nexus/TM3 interface and the upper nexus/TM3 interface, as outlined below.

The Orai1 channel pore is highly selective for Ca^2+^ and distinguished by its exceptionally low unitary conductance [157,158]. Its architecture consists of several distinct regions that collectively contribute to the maintenance of high Ca^2+^ ion selectivity and permeation. This includes the extracellular Ca^2+^-accumulating region (CAR) at the pore entrance [152], the selectivity filter (E106) [159–162], the hydrophobic central pore [162–166] and the intracellular basic region [149]. Together, these domains play a pivotal role in Ca^2+^ permeation through the channel [149,162,163].

## STIM1-Orai1 coupling

Direct interactions between STIM1 and Orai1 are crucial for Orai1 activation and the initiation of Ca^2+^ influx [26,52–54,59–61,167–173]. All three cytosolic regions of Orai1, the N-terminus, loop2, and C-terminus, are supposed to include potential contact sites for binding to the C-terminus of STIM1. While the role of the Orai1 C-terminus in coupling with STIM1 C-terminal CC domains is well established through structural and functional studies [54,55,155,174,175], the involvement of the N-terminus and loop2 remains a topic of debate [176–178]. Two prevailing hypotheses have emerged to explain the STIM1-mediated Orai1 activation mechanism. The first suggests that interactions between the STIM1 C-terminus and the Orai1 C-terminal segment alone are sufficient for channel activation [54–56,141,171]. The second proposes that additional contacts with the Orai1 N-terminus and loop2 are also required, potentially reflecting a sequential activation mechanism [61,153,155,178–183].

**Figure 2.**
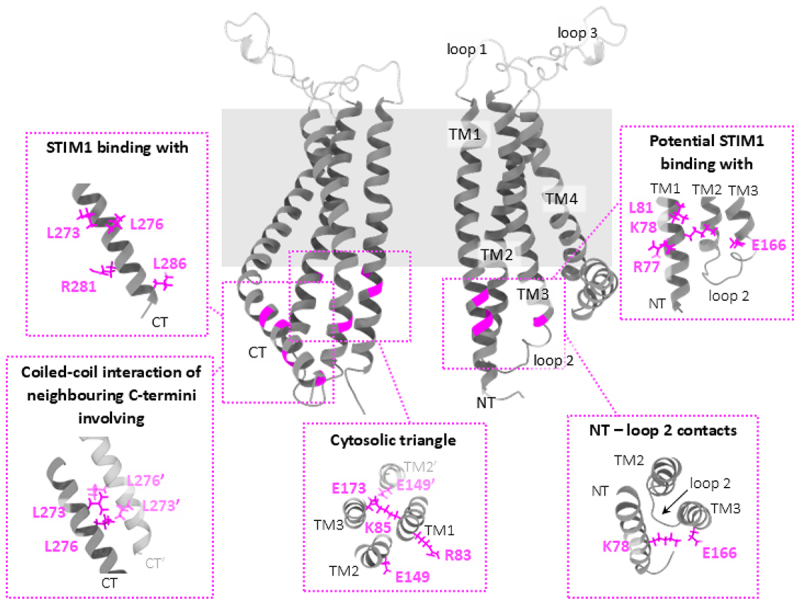
Critical sites within the cytosolic regions of the Orai1 channel.

**Figure 3.**
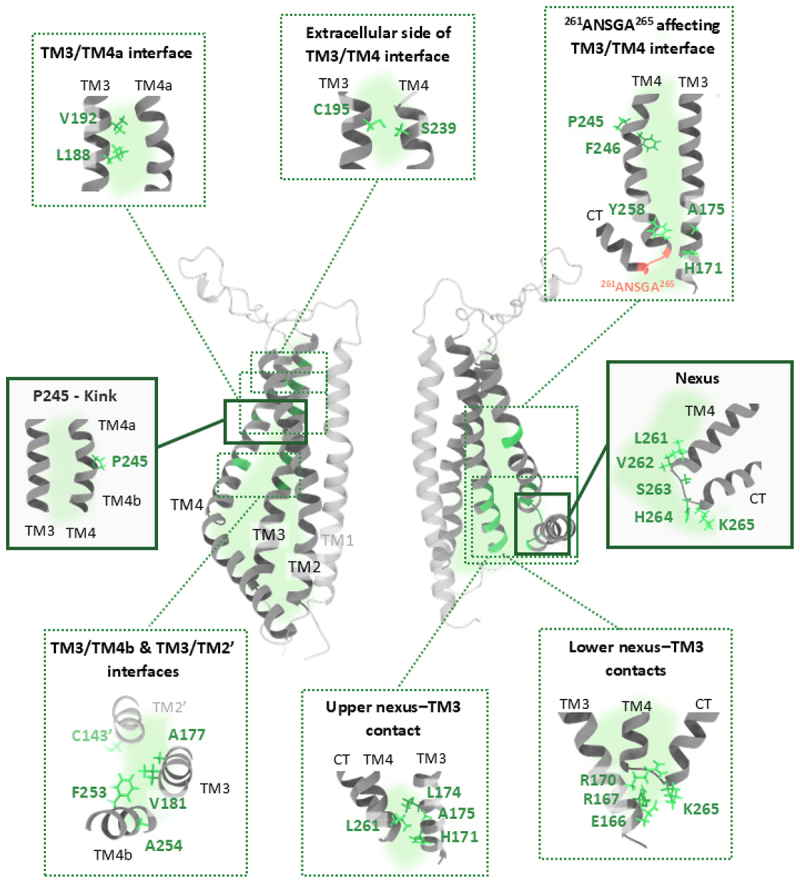
Critical sites within the outer interface of the Orai1 channel.

In both hypotheses, the Orai1 C-terminus emerges as the most critical interface for STIM1 interaction. Biochemical studies reveal strong binding between the CAD fragment of STIM1 and the Orai1 C-terminus [61]. Functional investigations uncovered L273 and L276 in the Orai1 C-terminus as critical hot spots for coupling to STIM1 [54](Figure 2, STIM1 binding with). These findings are in accord with the NMR structure of the STIM-Orai association pocket (SOAP), which is composed of two STIM1 C-terminal fragments (aa312-387) in complex with two Orai1 C-termini (aa 272–292), thus, forming the STIM1-binding interface in Orai1. It consists of two antiparallel CC regions formed by the C-termini of adjacent Orai1 subunits [141], which are stabilized primarily by hydrophobic contacts, involving L273 and L276 residues (Figure 2, coiled-coil interaction of neighboring C-termini involving; Table 1). Mutations of these residues to polar/charged amino acids (L273S/D, L276S/D) or truncation of this region disrupt STIM1 binding, likely due to reduced CC stability [54–56,171,179]. Additional residues, including R281, L286, and R289, further contribute to STIM1-Orai1 coupling [141](Figure 2, STIM1 binding with; R289 is not included in the structure; Table 1). Beyond the critical role of Orai1 C-terminal residues in STIM1 binding, mutations in the nexus region (S263W, K265G/W) led to reduced STIM1 coupling, whereas proline substitutions (S263P, K265P) markedly impaired STIM1–Orai1 assembly [180,184]. In line with these findings, we also uncovered a critical role for charged residues at the lower nexus/TM3 interface (described in more detail below) in facilitating STIM1 coupling to Orai1 and STIM1-mediated channel activation [185] (Figure 3, lower nexus–TM3 contacts; Table 2). pKa analyses using PROPKA 3 package [186,187] and a Monte-Carlo-based titration procedure based on the linearized Poisson-Boltzmann equation (MC-PBE) [188–190] revealed that residues E166, R167, H264, and K265 strongly favor their charged states at physiological pH, suggesting salt bridge formation in the closed state. Alanine substitutions of these charged residues showed that E166 and H264 are essential for STIM1-induced currents, whereas K265, R167, R170, and H171 appeared dispensable when mutated individually. Double charge-swap mutants (R167E K265E, R170E K265E, E166R H264R) exhibited reduced currents and FRET signals, confirming disrupted STIM1 coupling, underscoring the critical role of charged residues at the lower nexus–TM3 interface in STIM1 binding and pore opening [185]. However, whether these residues interact directly with STIM1 or exert their effects indirectly remains unclear, and further investigation is warranted.

Cross-sectional view of the hexameric Orai1 channel displaying two opposing subunits, with key cytosolic sites in the backbone highlighted in magenta. The rectangular background indicates the TM domain region embedded within the lipid bilayer of the plasma membrane. Insets show the side chains of the key cytosolic sites, grouped by their functional role or location: STIM1-binding with L273, L276, R281, and L286. Coiled-coil interactions of neighboring C-termini involving L273 and L276 from one subunit, and L273′ and L276′ (light magenta) from the adjacent subunit. The C-terminal region of the neighboring subunit (CT′) is shown in light gray. Cytosolic triangle: R83, K85, E149, E149′, and E173. TM2′ of the neighboring subunit is shown in light gray, with E149′ highlighted in light magenta. Potential STIM1 binding with R77, K78, L81 (NT), and E166 (loop 2). NT–loop2 contacts: K78 and E166. Some insets show the TM domains rotated by specific angles to enhance visualization of the highlighted residues.

Cross-sectional view of the hexameric Orai1 channel displaying two opposing subunits. TM4, TM3, and TM2 forming the outer interface (green-shaded region) are shown in dark gray, while the nonparticipating TM1 is depicted in light gray. Critical residues in the backbone along the outer interface are highlighted in green. Insets show the side chains of these residues, grouped by their functional role or location: TM3/TM4a interface: V192 and L188. P245–kink: P245. TM3/TM4b & TM3/TM2’ interfaces: C143,’ A177, V181, F253, and A254. TM2′ of the neighboring subunit is shown in light gray, with C143’ highlighted in light green. Extracellular side of TM3/TM4 interface: C195 and S239. Upper nexus–TM3 contact: H171, L174, A175 and L261. ^261^ANSGA^265^ affecting TM3/TM4 interface: H171, A175, F246, P245, and Y258. Nexus: ^261^LVSHK^265^. Lower nexus–TM3 contacts: E166, R167, R170, and K265. Some insets show the TM domains rotated by specific angles to enhance visualization of the highlighted residues.

**Table 1.** Crucial residues within Orai1 cytosolic regions (C‑terminus, loop2, and N‑terminus). Summary of critical residues in Orai1 cytosolic regions, associated gain‑of‑function (GoF) and loss‑of‑function (LoF) mutations, and currently known mechanisms versus unknown mechanisms and open questions.

Region	Residues	GoF mutation	LoF mutation/reduced activity	Known mechanism/notes	Unknown mechanism/open questions	Ref
C-terminus	L273, L276			Hot spots for STIM1-Orai1 coupling	Physiologically relevant conformational changes of the Orai1 C-termini in the Orai1 open and closed states	[54–56,171,179]
			L273S/D, L276S/D	Disrupt STIM1 binding		
	R281, L286, R289			Contribute to STIM1-Orai1 coupling		[141]
N-terminus	aa 70–91			Direct binding with CAD domain of STIM1	Mapping direct interaction between the STIM1 C-terminus and Orai1 N-terminus and/or loop2. Structural details of the N-terminus and loop2 are still awaited.	[61,179]
	R77, K78, L81			Potential hydrophilic binding interface for STIM1		[181]
	aa 1–78			Deleting this segment renders the channel nonfunctional		[55,153,179]
Loop2	E166	E166C		Potential interplay with the α3 domain of STIM1		[182]
N-terminus-Loop2	Y80-S152/N156, L79-L157/S159			Inhibitory interactions between these residues in loop2 and N-terminal in del 1–78		[178]
		Y80A/N156G		Disrupt inhibitory contacts between N-terminus and loop2		
	K78-E166		K78C E166C	Close sites; Crosslinking reduces activity		
	R83-E149, K85-E173, K85-E149			Crucial intra- and inter-subunit salt bridges for channel activation and stability (cytosolic triangles), at least two intact salt bridges are required for proper STIM1-mediated activation	Uncertain functional relevance of R83–E149 contact. Role of salt bridges in Orai1 activation/gating remains incompletely understood	[58,192]
				Orai1 gating involves a clockwise rotation of R83 to get closer to E149		
			K85E, E149K	Disrupt salt bridge interactions impairing channel function

**Table 2.** Crucial residues along the Orai1 outer interface. Summary of critical residues located in the four key structural regions of the outer interface (nexus, nexus–TM3 interface, non‑pore‑lining TM interface, and P245), their associated gain‑of‑function (GoF) and loss‑of‑function (LoF) mutations, relevance in disease, and currently known mechanisms versus unknown mechanisms and open questions.

Inter-face	Region	Residues	GoF mutation	LoF mutation/reduced activity	Disease related	Known mechanism/notes	Unknown mechanism/open questions	Ref
Outer interface	Nexus	^263^SHK^265^				Role in positioning the C-terminus for effective STIM1 binding		[180,184]
		^261^LVSHK^265^				Critical regulator to switch the channel between open and closed states	The overall role of the structure or flexibility of the nexus in Orai1 gating	[155]
			^261^ANSGA^265^			Mimic STIM1 activation and exhibit high Ca^2+^ selectivity	The mechanism by which ANSGA activates Orai1 is incomplete	
				S263P/W, K265P/G/W		Reduce or impair STIM1-Orai1 interaction		[180,184]
	Nexus-TM3 interface	L261-H171/L174/A175				Hydrophobic interactions regulate STIM1 binding and pore hydration	Unidentified functionally relevant contact sites between the nexus and TM3, and the identification of critical factors mediating these interactions, such as hydrophobicity.	[148,155]
				L261D/K, L174D/K, A175D/K		Reduced or abolished STIM1-induced activation		[155]
			L261C L174C			Enhances STIM1-induced gating after disulfide crosslinking		
			H171Azi, L174Bpa			UV-induced activation		[185,200,201]
		K265-E166/R167				Widening at the nexus-TM3 interface associated with STIM1-induced activation		[185]
			K265Azi/Bpa, R167Azi/Bpa			Crosslinking reduced channel activity		
			K265BetY/BprY/BptY E166C, K265BptY R167C					
			K265C E166C, K265C R167C					
		K265, H264, R167, R170, H171				Charged residues crucial for STIM1-binding and pore opening		
			E166A, H264A			Reduce STIM1 activation due to loss of or swapped key charges		
			R167E K265E, R170E K265E, E166R H264R					
	non-pore-lining TM interface	A175-Y258				Increased coupling between these residues in ANSGA mutant	Understanding the relationship between the nexus and TM4/TM3 contacts in transducing gating-relevant conformational changes toward the central pore	[212]
		H171, F246		H171Y, F246V		Alter α-helicity and local TM3-TM4 coupling in ANSGA context		
		C143, A177, V181, F253, A254				Potential widening at these interfaces is associated with pore opening	The functionally relevant interactions of these sites that maintain the open state still need to be identified. The function relevant role of the hydrophobic cluster of bulky residues (L174, V181, I182, F250, F253, F257) at the TM3/TM4 interface still not known.	[199,201]
			V181K, A254K			Charge-induced enhanced hydration in the pore, due to swelling of the channel complex, leading to pore widening.		
			C143W V181K, F253W V181K			Enhanced activation likely due to further expansion of this interface		
				V181F		Hydrophobic substitution leading to dewetting the TM3/TM4 interface		
			A177Azi, V181Azi/Bpa, A254Azi			UV-induced activation is possibly due to induced expansion at the non-pore lining interface		[200,201]
		L188, V192	L188Azi/Bpa, V192Azi/Bpa			UAA insertion widens the upper TM3/TM4 interface; crosslinking brings them closer	The contact sites in TM4 still need to be identified	[201]
		C195-S239				Oxidation forms sulfinic acid; enables H-bond with S239 stabilizing closed state		[220]
				C195D		Oxidomimetic mutant; mimics oxidation; persistent H-bond with S239		
			C195D S239A			Disrupts H-bond; restores channel activation above wild-type levels		
	P245		P245X; X: any amino acids			Proline interduces helix bend, stabilizes channel closure		[149,150,213]
			P245L		Stormorken-like syndrome			[92]

Although the role of STIM1 interactions with the Orai1 N-terminus and loop2 remains under dispute, both regions have been shown to play essential roles in STIM1 binding and STIM1-mediated channel activation. Biochemical analyses indicate that the CAD domain of STIM1 directly binds to the Orai1 N-terminal region, Orai1_70–91_ [61](Table 1). Supporting this, strong CAD binding is reported to Orai1_48–91_ and Orai1_68–91_, but not to the shorter fragment (Orai1_48–70_). Interestingly, the binding was stronger with the truncated Orai1_48–91_ construct than with the full-length N terminus, suggesting that residues 1–48 may negatively regulate CAD affinity [61]. This hypothesis is further supported by findings from McNally et al. [179], demonstrating that deletion of amino acid stretches 1–85 or 73–85 in Orai1 results in a 50% reduction in CAD binding. Förster resonance energy transfer (FRET)-derived interaction in a restricted environment (FIRE) and biochemical experiments demonstrated further that STIM1 C-terminal segments interact with the Orai1 N-terminus [61,181] and loop2 [178]. Wang et al. [120] proposed a potential interaction between hydrophobic residues located in the distal region of the Orai1 N-terminus and F394 within the SOAR domain of STIM1 [120]. Contrastingly, more recent findings by Niu et al. [181], using STIM1 fragments and synthetic peptides, propose a hydrophilic binding interface involving residues R77, K78, and L81 in the Orai1 N-terminus (Figure 2, potential STIM1 binding with; Table 1). Notably, the affinity of STIM1 to the Orai1 C-terminus appears to be stronger than its affinity for the N-terminus [61,180,181,191]. However, as these findings were based on studies using truncated fragments, further validation with longer segments or ideally, full-length Orai1 is imperative to confirm these interactions under more physiological conditions.

Furthermore, intra- and inter-subunit salt bridges (R83-E149, K85-E173, K85-E149; (Figure 2, cytosolic triangle; Table 1) formed between the N-terminus and the loop2 segment have been identified by MD simulation and are outlined below in more detail in terms of conformational dynamics within the channel complex. In addition to their modulatory role within the Orai1 complex, they play a critical role in stabilizing STIM1 coupling as revealed by functional studies [58,192].

Within the loop2 region of Orai1, Butorac et al. [182] identified a potential interplay between residue E166 and the α3 domain of STIM1, a short helical segment spanning aa 400–403. This close proximity was validated through cysteine crosslinking experiments, in which diamide application led to increased currents. This indicates that the physical association between STIM1 (around residue L402) and Orai1 (near E166) is crucial for channel gating (Figure 2, potential STIM1 binding with; Table 1).

Overall, the Orai1 C-terminus serves as the primary site for direct coupling with the C-terminus of STIM1. However, it remains unclear whether the N-terminus and cytosolic loops of Orai1 also function as direct interaction partners. Subsequent engagement of the N-terminus and loop2 may occur following initial C-terminal binding, suggesting a sequential mechanism of STIM1 association that contributes to channel activation. Although functionally relevant interaction sites within the cytosolic regions of Orai1 have been reported, the precise contact partner residues in STIM1 mediating its binding remain incompletely defined.

## Stoichiometry of the STIM1–Orai1 complex

In addition to identifying key functionally relevant binding sites, the stoichiometric requirements governing STIM1-Orai1 association remain an active area of investigation [62,141,193–197], with three major models currently proposed. The bimolecular binding model [141], supported by nuclear magnetic resonance (NMR) studies, suggests that a dimer of STIM1 C-terminal fragments binds directly to the antiparallel-oriented C-termini of Orai1 in a 1:1 STIM1:Orai1 ratio. The unimolecular model [62], based on fluorescence recovery after photobleaching (FRAP) and super-resolution microscopy, proposes a global 1:1 stoichiometry. In this model, each monomer of a STIM1 C-terminus dimer interacts with a subunit of two distinct hexamers, effectively bridging two neighboring Orai1 channels and potentially promoting channel clustering. The sequential step model [195] suggests a 2:1 STIM1:Orai1 ratio for maximal activation. Here, one strand of a STIM1 dimer is assumed to initially bind an Orai1 subunit, inducing structural rearrangements that enhance its affinity for the second monomer. Full activation requires both strands to engage, implying that 12 STIM1 molecules are needed to fully activate a hexameric Orai1 channel. Studies using concatemeric Orai1 constructs with the L273D mutation (Table 1) have provided valuable insights into cooperativity and functional stoichiometry [198]. Mutation of just one out of six C-terminal STIM1 binding sites lowered the opening probability (Po) of the channel by approximately 90%, demonstrating a non-linear relationship between STIM1 binding and Orai1 activation. Additionally, this single-subunit mutation impaired Ca^2+^ selectivity, increased single-channel conductance, and reduced Na^+^ over Cs^+^ permeability. These findings highlight the critical role of STIM1 binding to all six subunits for proper CRAC channel function. Interestingly, modeling of CRAC channel activation as well as free energy calculations based on monomeric stability of STIM1, indicate a negative cooperativity of STIM1 binding to Orai1, with a cooperativity coefficient of 0,5 [142,193]. There is a clear preferential interaction of a STIM1 dimer to activate one functional unit of Orai1, a hexamer. This indicates that binding of STIM1 dimer induces conformational rearrangement not only of the bound, but also of the unbound C-termini, making the binding of additional STIM1 dimers less probable. However, it remains unclear whether binding of a single STIM1 dimer is sufficient for full activation of the Orai1 hexamer, as a single L273D within an Orai1 concatemer almost fully abrogates STIM1-mediated Orai1 activation [198].

Collectively, these findings emphasize the intricate nature of STIM1-Orai1 interactions, highlighting both structural and functional roles in channel activation. Further investigation is needed to explore whether distinct stoichiometric configurations correspond to unique activation states of the channel, potentially leading to diverse signaling outcomes in different physiological contexts.

## Sequential signal propagation from the Orai1 C-terminus at the periphery to the pore in the center

Extensive efforts have been undertaken to elucidate the dynamic behavior of Orai channels, employing a wide array of approaches, including structural analyses [149–151,192,199], conventional mutagenesis [27,58,151,155,164,165,178,180,183,199–208], molecular modeling [192] and genetic code expansion [185,200,201]. Structural studies have provided valuable insights into the closed and open conformations; however, these conformations were resolved in the absence of STIM1. Consequently, the precise structural rearrangements triggered by STIM1 binding that culminate in pore opening remain incompletely understood. Pore dilation upon Orai1 activation is well established [124,148–151], yet the underlying mechanism driving this expansion remains a topic of debate. Comparative structural analyses of the closed and open states suggest that global conformational rearrangements across all TM domains play a key role in channel opening [148–151]. Recent cryo-EM analysis of the dOrai H206A has shed light on this process, revealing a rigid-body outward movement of subunits during activation [151]. Notably, this movement occurs without additional conformational changes, such as rotation. In contrast, MD simulations propose a twist-to-open gating mechanism, wherein the TM1 helices undergo counterclockwise rotation on the extracellular side, facilitating pore dilation. Concurrently, on the intracellular side, three subunits rotate clockwise while the remaining three move outward [192]. Moreover, targeted MD studies provided first insights into the formation and break of a series of contact sites upon the transition from the closed to open state and vice versa [209].

Overall, these studies indicate clear global conformational changes across the entire channel complex that facilitate pore opening [57,58,154,210]. This mechanism is further supported by various functional studies and TM domain scanning experiments [57,58,151,155,164,165,178,180,183,200–203,205–208,211]. Importantly, these conformational changes are not confined to the TM domains but also extend to both intra- and extracellular regions, as will be discussed in greater detail in the following sections. Despite these advances, key uncertainties persist.

In the following sections, we delve into the latest findings regarding Orai channel dynamics and the currently known critical molecular transitions that govern its activation. Since Orai1 pore opening is initiated by STIM1 coupling at the C-terminus, we will first start with the current insights into the dynamics at the cytosolic regions and then continue with those of the TM domains. Special emphasis will be given to the dynamic behavior of key residues, potential contact sites and TM domains located at three pivotal interfaces: the outer interface (between TM4 and TM2/3), the inner interface (between TM2/3 and TM1), and the pore (Figure 1(a-c)). Additionally, we will highlight the roles of the extracellular regions in modulating channel gating.

## Structural rearrangements of cytosolic regions

### Orai1 C-terminus

Based on the currently available closed and open state structures, it remains unclear which structural changes along the C-termini are energetically favorable under physiological conditions in both STIM-free and STIM-bound states. In the closed structure, the C-termini of adjacent subunits adopt an antiparallel arrangement, with the CC domains forming a belt-like structure around the channel [149]. In contrast, open structures using crystallography, such as those of dOrai H206A (PDB: 6BBF) and P288L (PDB: 6AKI) mutants, reveal a complete straightening of the TM4 segments and C-termini through unlatching conformational changes [148,151]. This unlatched state has been proposed as a critical step for channel activation. However, the X-ray structure of the LoF mutant Orai1 R91W exhibits pore widening in the basic region but shows a straightened TM4 C-terminus [150]. This observation suggests that conformational changes along the TM4 C-terminal segment alone are likely insufficient to drive pore opening. Furthermore, it remains unclear to what extent C-terminal conformational changes occur upon STIM1-coupling. Cysteine crosslinking experiments revealed that disulfide bond formation between adjacent C-termini prevents maximal STIM1-mediated Orai1 activation [184]. Cryo-EM structures (cryo-EM; PDB: 7HR5) suggest less pronounced structural changes along the TM4 C-terminal region compared to those observed in open X-ray structures [148,151]. Hence, one prevailing hypothesis suggests that the Orai1 C-termini undergo minor conformational changes during STIM1 binding, sliding against each other to interact with the STIM1 SOAR/CAD domain [141,184,195]. Nevertheless, further investigations are needed to understand the interplay of structural rearrangements required for channel activation (Table 1).

### Orai1 N-terminus and loop2

Structural data of the closed Orai crystal structure [149] suggest that the N-terminus is shielded in its resting state, implying that significant conformational rearrangements are required to expose the N-terminus for STIM1 binding and subsequent pore opening. Truncation of the N-terminus (ΔN1–78) renders the channel nonfunctional [55,153,179](Table 1). MD simulations of hexameric Orai1 [178] revealed that this LoF can be attributed to a reorientation of loop2, as evidenced by an increase in root mean square deviation (RMSD). This structural shift causes new inhibitory interactions between loop2 and N-terminal residues (L79–L157/S159; Y80–S152/N156; Table 1), effectively preventing channel activation, an effect not observed in Orai3. Notably, disrupting these interactions through specific mutations (Y80A/N156G) restored Ca^2+^-selective currents in the presence of STIM1 [178]. Moreover, cysteine crosslinking between the N-terminus (K78C) and loop2 (E166C) (Figure 2, NT–loop2 contact; Table 1) in full-length Orai1 demonstrated that close proximity of these regions leads to a significant reduction in store-operated currents. This finding highlights the inhibitory interactions between the N-terminus and loop2, which can negatively impact channel activation.

Additionally, MD simulations on the closed-state Orai1 structure revealed that the positioning and interactions of critical residues in the N-terminus (R83, K85) and loop2 (E149, E173) [58] (Figure 2, cytosolic triangle; Table 1) are crucial for Orai1 function. The simulations identified potential salt-bridge interactions between K85-E173 and K85-E149, which are crucial for Orai1 stability and activation. In contrast, the R83–E149 interaction was less stable and only occurred when TM1 exhibited slight flexibility, allowing R83 to rotate toward E149. Mutations disrupting these salt-bridge interactions (e.g. K85E and E149K) led to pore collapse and reduced hydration, severely impairing channel function. However, compensatory mutations (e.g. L81K) restored pore integrity and channel activity, emphasizing the importance of these salt-bridge interactions [58] in maintaining the structural and functional integrity of the Orai1 channel. These findings align with the proposed open-state model, which suggests that Orai1 gating involves a clockwise rotation of R83, bringing it closer to E149 [192]. Disruption of these salt bridges significantly impairs Orai1 activation, with at least two intact salt bridges required for proper STIM1-mediated channel activation [58]. However, the functional significance of the R83-E149 salt bridge remains uncertain. Structural data from both the closed and open dOrai structures [149], show that R83 and E149 are approximately 14 Å apart, with R83 oriented toward the central pore (Figure 2, NT–loop2 contact; Table 1). This spatial arrangement might hinder a direct interaction between R83 and E149, raising questions about the precise role of this salt bridge in Orai1 gating and activation. Further studies are needed to clarify its contribution to channel function.

In conclusion, these findings underscore the intricate interplay between the Orai1 N-terminus, loop2, and STIM1 in orchestrating channel activation. The coordinated interaction between the N-terminus and loop2 likely forms a functionally indispensable STIM1-binding pocket. Several salt-bridge interactions appear to contribute to the precise communication between the N-terminus, loop2, and the STIM1 C-terminus, facilitating effective channel gating.

Overall, a plausible scenario within the intracellular regions is that the C-termini adopt an antiparallel configuration in the inactive state. Upon STIM1 binding, both the C-termini and TM4 helices undergo straightening, disrupting the compact TM4/TM3 interface (see below). This structural rearrangement likely induces an outward displacement of all TM domains, promoting pore dilation and channel opening. Concurrently, TM4 straightening may reposition loop2 away from the N-terminus, potentially unmasking the N-terminal region for STIM1 interaction. Such repositioning could also facilitate expansion of the basic region adjacent to the pore, further enhancing channel activation and ion conduction.

## Structural rearrangements along the outer interface (between TM4 and TM2/TM3)

Following STIM1 coupling, the gating signal is transmitted through the nexus region [155] via conformational changes within the nexus itself and potential interplay with TM3, ultimately propagating across the entire channel complex. Moreover, the outer interface (Figure 1, b-c) serves as a crucial element for conferring the gating signal within the membrane. Accordingly, numerous studies have explored the structural dynamics and interactions underlying Orai pore opening, with particular emphasis on the TM3/TM4 interface [58,64,92,155,199,212,213]. Along the outer interface, we will highlight the known dynamics and mechanisms at key structural regions: (1) the hinge or nexus region [155], (2) the nexus–TM3 interface, (3) the non-pore lining TM interfaces, and (4) the P245 kink located centrally within TM4 [148]. Together, these regions play a critical role in transmitting conformational changes that govern Orai1 channel gating.

### The nexus region

Notably, a sharp kink (^263^SHK^265^) links the C-terminal extensions to TM4, disrupting the continuity of these α-helices. This structural bend is thought to play a critical role in positioning the C-terminus for effective STIM1 binding [180,184]. Potential motion of the C-termini likely relies on the flexibility of the kink. Structural investigations using crystallography [150] show a straightening of this kink in the open compared to the closed state of Orai. Zhou et al. [155] proposed that the ^263^SHK^265^ motif serves a dual role in STIM1 binding and channel gating. This segment is part of the so-called nexus region (^261^LVSHK^265^) (Figure 3, Nexus; Table 2), identified as a critical regulator of the channel to switch between open and closed states [155]. While single mutations in ^263^SHK^265^ disrupt STIM1 binding [180,184], replacement of the ^261^LVSHK^265^ with ^261^ANSGA^265^ renders the channel constitutively active. These mutations induce an activation state resembling that triggered by STIM1 and exhibit high Ca^2+^ selectivity [155], further underscoring the regulatory importance of this region. Recently, Augustynek et al. [212] conducted further investigations to elucidate how the ANSGA mutant transmits gating signals from the distal TM regions to the pore. Helicity analysis revealed that the ANSGA mutation enhances α-helicity of the LVSHK nexus region of Orai1 but does not affect the TM4 proline bend at P245 (will be described later) (Figure 3, ^261^ANSGA^265^ affecting TM3/TM4 interface; Table 2).

Structural screening and contact analysis further demonstrated that ANSGA mutations affect the contact frequency of TM3 (A175) and TM4 (Y258) (Figure 3, ^261^ANSGA^265^ affecting TM3/TM4 interface; Table 2). Notably, while ANSGA triggered constitutive activation in Orai1, it failed to activate Orai2 and Orai3. This finding indicates that specific structural features unique to Orai1 are required for ANSGA-mediated activation. A key factor appears to be a residue variation, as H171 in the TM3 extension and F246 in TM4 are not conserved across Orai paralogs. Substituting H171 and F246 in Orai1 with the corresponding tyrosine and valine residues found in Orai2 (Y145, V207) and Orai3 (Y146, V255), as well as dOrai (Y250, V303) abolished ANSGA‑induced constitutive activation of Orai1 (Orai1 H171Y‑ANSGA, Orai1 F246V‑ANSGA) and also prevented STIM1‑dependent activation. In contrast, STIM1‑mediated gating of the single mutants remained unaffected for Orai1 H171Y and was only slightly reduced for Orai1 F246V (Figure 3, ^261^ANSGA^265^ affecting TM3/TM4 interface; Table 2). In the same study, the authors also showed that although the LVSHK motif is fully conserved in *Xenopus laevis* Orai1 (xOrai1), mouse Orai1 (mOrai1), and dOrai, only the xOrai1‑ANSGA mutant displayed remarkable constitutive activity, whereas mOrai1-ANSGA and dOrai‑ANSGA remained inactive. Introducing the F246C mutation into Orai1‑ANSGA (corresponding to C249 in mOrai1) abolished its constitutive activity. Conversely, the mOrai1‑ANSGA mutant became constitutively active only when the C249F substitution was introduced to mimic the Orai1 F246 residue. This highlights the critical role of F246 in Orai1 in relaying conformational changes along the gating pathway. MD simulations further support these observations, showing that the H171Y and F246V mutations in the ANSGA-Orai1 channel alter the α-helicity of the TM4 helix extension (residues 259–269) and alter local TM3-TM4 coupling [212]. These findings underscore the importance of specific residue interactions and structural features in mediating ANSGA-induced activation and highlight the unique gating properties of Orai1 compared to its paralogs.

### The nexus-TM3 interface

The nexus region directly connects to the TM3/TM4 interface on the cytosolic side. Thus, it has been proposed to serve not only in positioning the C-terminus for effective STIM1 binding but also as the initial structural bridge linking the STIM1-binding site in the Orai1 C-terminus to the TM domains. Structurally, this bridge comprises two distinct segments: a lower flexible portion (K265, H264, S263; Figure 3, lower nexus–TM3; Table 2) and an upper hydrophobic portion (V262, L261; Figure 3, upper nexus–TM3; Table 2), both of which have been shown to play critical roles in channel gating.

At the lower nexus, we recently observed that widening at the lower nexus–TM3 interface (Figure 3, lower nexus–TM3; Table 2) is essential for proper STIM1-induced Orai1 pore opening, as evidenced by markedly reduced activation upon crosslinking at this interface. In our attempt to probe the dynamics and binding interfaces at single amino acid resolution with high spatiotemporal precision, we employed the genetic code expansion (GCE) technique [214–216] to incorporate unnatural amino acids (UAAs) at key positions within this interface [185]. Specifically, we introduced photocrosslinking UAAs, p-benzoyl-L-phenylalanine (Bpa) and p-azido-L-phenylalanine (Azi). Upon UV irradiation (365 nm), these UAAs become reactive and form covalent bonds with nearby X – H moieties (X = C, N, S, O) within a 3–4 Å radius [217,218]. In addition, we utilized the chemical crosslinking UAA BCnY, which spontaneously and covalently reacts with proximal cysteine thiolates [217,219]. Using the approach with photocrosslinking UAAs, UV light exposure reduced STIM1-induced activation of Orai1 K265Azi/Bpa, likely due to crosslinking with TM3 residues. Moreover, applying UV light prior to STIM1 binding hindered subsequent STIM1-induced activation of Orai1 K265Azi/Bpa. Comparable effects were further observed by inserting photocrosslinking UAAs at the oppositely located site in TM3, R167 (Orai1 R167Azi/Bpa). Insertion of chemical crosslinking UAAs at these sites combined with a cysteine at an opposite position (K265BetY/BprY/BptY E166C, K265BptY R167C), also led to a marked reduction or complete loss of STIM1-induced activation. This highlights the importance of specific contacts at the lower nexus–TM3 interface in maintaining the closed state of the channel. We further supported this conclusion with cysteine-crosslinking experiments involving K265C and E166C/R167C (Figure 3, lower nexus–TM3; Table 2). The formation of a disulfide bond between these cysteines significantly reduced STIM1-induced channel activation [185]. In addition to the observed widening at this interface, residues with charged side chains (E166, R167, H264, K265; Table 2) play a critical role in maintaining STIM1-mediated Orai1 activation [185]. However, at this stage, it remains unclear whether these charged residues affect STIM1 binding directly or indirectly and/or STIM1-induced pore opening dynamics.

In extension, the first hot spot in the conformational cascade downstream the nexus is the LV-hinge plate (V262, L261) in the upper nexus region (Figure 3, upper nexus–TM3). Hydrophobic interactions with TM3 have been shown to play a vital role in locally relaying the conformational transition toward the open state [155]. A key structural connection represents L261 (TM4) and L174/A175 (TM3), playing a dual role in regulating both STIM1 binding and pore hydration [148,155](Figure 3, upper nexus–TM3; Table 2). Substituting these residues with charged amino acids significantly reduced (L261D/K) or completely abolished (L174D/K; A175D/K) STIM1-induced activation. Interestingly, STIM1 coupling remained partially intact in L174D/K mutants [155], suggesting a degree of functional resilience. Consistently, MD simulations revealed a dewetting effect in the hydrophobic region of the pore in the GoF mutant Orai1 H134A containing the L174D substitution [58]. Disulfide crosslinking between Orai1 L174C and Orai1 L261C enhanced STIM1-induced gating, reinforcing the significance of the upper nexus-TM3 interplay [155]. Additionally, Liu et al. [148] demonstrated that these hydrophobic interactions, along with close-by sites (F178A (TM3) F257A/TM4) at the TM3/TM4 interface, are indispensable for Orai1 activation. Our MD simulations of membrane-embedded Orai1 to analyze inter-TM and inter-residue contacts revealed an almost 100% contact frequency between L174 in TM3 and residues in TM4, likely L261 [201], underscoring a tightly packed interface. Building on this observation, we incorporated photocrosslinking UAAs at key positions at the upper nexus–TM3 interface, specifically at Orai1 H171 (H171Azi) and L174 (L174Bpa). In the absence of STIM1, neither Orai1 H171Azi nor Orai1 L174Bpa exhibited notable channel activity prior to UV exposure. However, upon UV application, only Orai1 L174Bpa showed robust channel activation, likely due to covalent crosslinking with TM4. In the presence of STIM1, both UAA-containing Orai1 variants allowed STIM1-mediated activation and exhibited further enhancement upon UV exposure. This effect is reminiscent of the previously reported hydrophobic gating interaction at this interface involving H171, L174, and L261 [200,201]. Yet, the physiologically relevant contact sites remain to be fully resolved. Notably, since Orai1 H171Azi shows UV-induced functional effects only in the presence, but not the absence of STIM1, this clearly indicates that STIM1-binding induces conformational changes around this area, making functionally relevant contacts for Azi at H171 available (Table 2).

Overall, the nexus region plays a critical role in transmitting the activation signal from the STIM1-binding site to the peripheral TM domains of the channel complex. Hence, elucidating the mechanistic details of how individual residues within the nexus interact with other TM domains, particularly TM3, will be essential for understanding the molecular basis of STIM1-mediated channel activation. Furthermore, investigating critical factors such as hydrophobicity that may influence this interplay could substantially advance our knowledge of Orai1 gating dynamics.

### The non-pore lining TM interfaces

Several critical residues (e.g. A235, S239, and F250) that contribute to the regulation of Orai1 pore opening have been identified at non-pore-lining TM interfaces [58,64]. Although their precise mechanisms and roles were initially unclear, recent studies have underscored their functional significance [58,148,155,185,199,201,209,212,220].

Our recent studies revealed that hydration at the channel periphery can influence the interplay of TM domains at the outer interface, and thereby modulate Orai1 function. Hopl et al. [199] showed that introducing charged residues in the lower segment of TM3 or TM4b domains, such as at V181 (V181K) in TM3 and the opposite positioned A254 (A254K) in TM4b, resulted in a significant increase in constitutive Orai1 activity (Figure 3, TM3/TM4b & TM3/TM2’ interfaces; Table 2). MD simulations on these charged mutants, especially Orai1 V181K, in comparison to wild-type Orai1, revealed increased equilibration times and greater overall structural flexibility. These altered structural dynamics correlate with an increased pore radius and enhanced hydration not only along the pore, but also at the TM3/TM4b interface, which is proposed to underlie the constitutive channel activation. Overall, these findings indicate that charge-induced enhanced hydration along the non-pore-lining TM interface causes swelling of the channel complex. This swelling enhances pore hydration and ultimately leads to pore widening. Consistently, targeted MD simulations investigating inter-helical distances revealed that TM3 and TM4b move apart, involving a radial motion of TM3 upon the transition from the closed to the open state [209]. Further functional analysis revealed that substituting residues near V181K with bulky amino acids, specifically C143W V181K (C143 in TM2 of the adjacent subunit, TM2′) and F253W V181K (F253 in TM4b) (Figure 3, TM3/TM4b & TM3/TM2’ interfaces; Table 2), enhanced currents of Orai1 V181K further, likely due to expansion of this region. In contrast, introducing hydrophobic aromatic residues one helical turn below V181K in TM3 (A177W V181K) or in TM4b (V181K F257W) abolished the constitutive activity of Orai1 V181K, presumably due to water shielding effects. Consistently, substitution of V181 with phenylalanine (V181F) also resulted in LoF, potentially by maintaining a dewetted TM3/TM4b interface. We further confirmed the functional importance of the residues surrounding V181K by mutating them to amino acids of varying sizes and hydrophobicity. These substitutions modulated the constitutive activity of Orai1 V181K. These findings align with our previous observations that reduced hydrophobicity along the TM3/TM4 interface promotes channel closure, a process governed in an isoform-specific manner by non-conserved gating checkpoints within TM3 [221]. Overall, these findings suggest that loss of hydrophobicity along the TM3/TM4 interface or even hydration-induced conformational changes, likely widening, of the TM3/4b interface, are crucial for Orai1 channel activation and such structural rearrangement might be mimicked by STIM1-induced pore opening [199].

To further investigate how a positively charged side chain in TM3 affects Orai1 gating and to gain deeper mechanistic insights into Orai1 channel dynamics at the non–pore-lining TM interface, we combined photocrosslinking UAA insertion (Azi, Bpa) with conventional site-directed mutagenesis. This comprehensive strategy allowed us to systematically probe how structural dilation at the TM3/TM4 and TM3/TM2′ interfaces relates to channel activation. More specifically, along the TM3/TM4 interface, two distinct outcomes emerged depending on the location of UAA insertion in TM3.

Insertion of the bulky UAAs (Azi, Bpa) at TM3 sites within the TM3/TM4a interface (e.g. L188Azi/Bpa, V192Azi/Bpa; Figure 3, TM3/TM4a interface; Table 2), led to constitutive channel activity, which was significantly reduced upon subsequent UV light application. MD simulations of wild-type Orai1 showed that residues in the TM3 segment within the TM3/TM4a interface form nearly 100% of their contacts with TM4. Accordingly, we propose that inserting bulky UAAs at the TM3/TM4a interface pushes TM3 and TM4a apart, potentially inducing pore opening, while UV-induced crosslinking brings the TM domains closer together, likely leading to channel closure.

In contrast, UAA insertions at TM3 sites within the TM3/TM4b interface exhibited little to no basal activity, but UV exposure triggered channel activation (e.g. Orai1 L174Bpa, Orai1 A177Azi, Orai1 V181Azi/Bpa, Orai1 A254Azi; Figure 3, TM3/TM4b and TM3/TM2′ interfaces; Table 2) [201]. MD simulations of wild-type Orai1 provided further insight, revealing that residues in the TM3 segment within the TM3/TM4b interface interact with either TM4b or TM2.’ Thus, we hypothesized that the bulkiness of the UAA at the TM3/TM4b interface seems not sufficient to induce pore opening, likely due to side chain orientation toward TM4b and TM2“. However, together with UV light, UAA incorporation enables the stabilization of TM3/TM4b or TM3/TM2” in a conformation that promotes pore opening [201]. Functional changes observed on key Azi/Bpa mutants at the TM3 segment within the TM3/TM4b and TM3/TM2′ interface were further examined by substituting their potential direct or indirect contact partners in TM4b (e.g. A254) or TM2′ (e.g. C143) with amino acids of varying side chain volumes. A clear correlation emerged, in which double mutants introducing increased side chain volume into the TM3/TM4b and/or TM3/TM2′ interfaces showed enhanced channel activity following UV irradiation. Despite the strong correlation between side chain volume and Ca^2+^ current activation, we found double mutants that deviate from this trend. Such exceptions probably occur due to the unfavorable orientation of the reactive group of the UAA or due to a deficiency of functional effects caused by photocrosslinking. Overall, we hypothesize that a combination of appropriate UAA side chain orientation, accessibility of the reactive group, and appropriate position in the channel complex are key determinants for the occurrence of UV-derived functional alterations, and to resolve distance-dependent effects along the TM3/TM4 interface [201].

**Figure 4.**
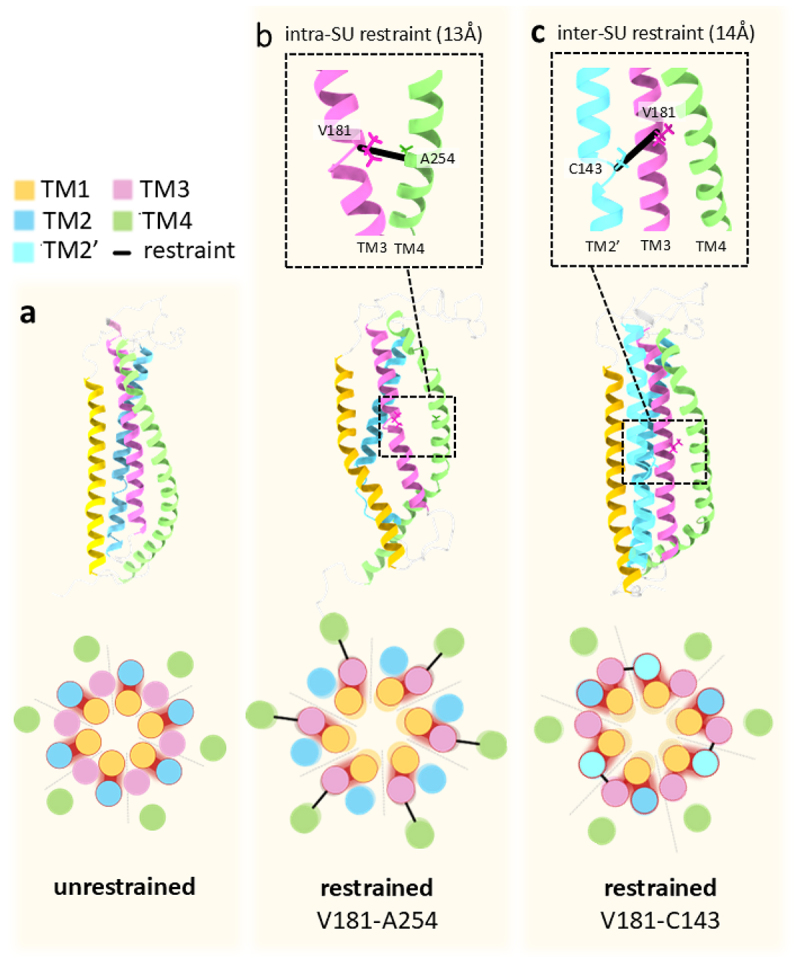
Increased distance between V181 and its opposing residues, A254 in TM3 or C143 in TM2,’ triggers Orai1 pore opening in MD simulations.

Biased MD analysis further supported this interface‑specific behavior: increasing the distance between V181 in TM3 and its opposing residues (A254 in TM4b or C143 in TM2′) enhanced pore hydration and dilation (Figure 3, TM3/TM4b and TM3/TM2′ interfaces; Table 2). Notably, in the unrestrained channel, all V181:A254 pairs exhibit a resting distance of approximately 6 Å, whereas only three of the six V181:C143 pairs are in close proximity (6–10 Å). Accordingly, in the TM3/TM4b d(V181:A254)‑restrained channel, restraints were applied to all V181:A254 pairs, while in the TM3/TM2’ d(V181:C143)‑restrained channel only the three pairs located in closer proximity were restrained, with the remaining three left flexible (Figure 4). In addition, compared to the closed state (unrestrained Orai1 channel), where TM2 serves as the primary interaction partner for TM1, TM1 shifts its interaction preference to TM3 in the TM3/TM4b d(V181:A254)-restrained channel, resulting in pore dilation. In contrast, in the TM3/TM2′ d(V181:C143)-restrained channel, TM2 becomes the dominant interaction partner for TM1, while its interaction with TM3 is comparatively weaker (Figure 4). Principal component analysis (PCA) [222] provided additional insights, revealing that restraining the V181-A254 distance at 13 Å enhanced both radial and tangential motion of TM1, while a 10 Å restraint enhanced tangential motion in one TM1 helix. Moreover, perturbation response scanning (PRS) [223,224] identified V181 and A254 as allosteric hotspots, highlighting their critical role in transducing mechanical signals to the pore. Interestingly, restraining the TM3-TM4b distance also increased fluctuations in the side-chain orientation of F99 within the hydrophobic cavity of the pore, suggesting a potential role for this residue in gating [201].

a) Unrestrained Orai1 subunit with TM1, TM2, TM3, and TM4 helices colored yellow, blue, magenta and green, respectively (top). Schematic representation of the unrestrained channel viewed from the extracellular side (bottom), where TM2 serves as the primary interaction partner of TM1. Red shaded areas indicate the interaction between TM1 and TM2. Subunits are separated by dashed lines. b) Restrained Orai1 subunit with TM1, TM2, TM3, and TM4 helices colored as in panel (a) (top). The inset shows intra-subunit restraint between residues V181 in TM3 and A254 in TM4, with d(V181:A254) set to 13 Å (black line). Schematic representation of the Orai1 channel viewed from the extracellular side under the Orai1-d(V181:A254)-restrained condition (bottom), where increased distance between TM3 (magenta) and TM4 (green) shifts the primary TM1 interaction partner from TM2 to TM3, resulting in pore dilation. Red shaded areas indicate the new interaction between TM1 (yellow) and TM3 (magenta). Restraints were applied to all six V181:A254 pairs. Subunits are separated by dashed lines. c) Restrained Orai1 subunit with TM1, TM2, TM2′ (TM2 of the neighboring subunit), TM3, and TM4 helices colored yellow, blue, cyan, magenta, and green, respectively (top). The inset shows an inter-subunit restraint between residues V181 in TM3 and C143 in TM2′, with d(V181:C143) set to 14 Å (black line). Schematic representation of the Orai1 channel viewed from the extracellular side under the Orai1-d(V181:C143)-restrained condition (bottom), where increased distance between TM3 (magenta) and TM2′ (cyan) enhances the interaction between TM1 and TM2, compared to TM3 (indicated by the thickness of the red shaded lines), resulting in pore dilation. Red shaded areas indicate the interaction between TM1 (yellow) and TM3 (magenta) and TM2 (blue, cyan). Restraints were applied exclusively to the three V181:C143′ pairs located in close proximity, whereas the other pairs were not restrained. Subunits are separated by dashed lines.

Since both hydration and increased steric dilation in the TM interfaces lead to CRAC channel-like pore opening already in the absence of STIM1, we hypothesized that the linkage between the expansion of the outer interface and pore opening is physiologically relevant. Indeed, for Orai1 mutants containing Azi/Bpa along the TM3/TM4 and TM3/TM2’ interfaces, UV light application after full STIM1 binding led to a significant increase in channel activity. Conversely, UV irradiation before complete STIM1 coupling restricted STIM1-mediated activation, likely due to covalent bond formation that locked the interfaces in a fixed conformation, preventing the necessary structural rearrangements for activation [201].

In extension, at the extracellular side of the TM3/TM4 interface, Alansary et al. [220] identified a thiol-dependent intramolecular locking mechanism that inhibits Orai1 activation under oxidative conditions (Figure 3, extracellular side of TM3/TM4 interface; Table 2). While STIM1-Orai1 coupling and FCDI remained unaltered upon H_2_O_2_ incubation, divalent free currents and Orai1 clustering were affected. MD simulations using a homology model of Orai1, based on dOrai1 and incorporating the CAD/SOAR domain of STIM1, were employed to assess gating and redox-sensitive interactions. Oxidation of C195 in TM3 elongates its side chain via sulfinic acid formation, reducing its distance to S239 from 2.7 Å to 1.6 Å. This enables a stabilizing hydrogen bond between the sidechain thiolate of oxidized C195 and the hydroxyl group of S239 (Figure 3, extracellular side of TM3/TM4 interface; Table 2). According to unrestrained blind docking, this interaction locks the channel in a closed conformation without affecting STIM1 binding. In the open state, this interaction is geometrically unfavorable, indicating that oxidation selectively stabilizes the closed state. Functional assays with the oxidomimetic mutant C195D revealed current inhibition, likely due to a persistent hydrogen bond with S239. A C195D S239A double mutant restored I_CRAC_ to levels slightly exceeding wild-type levels. This confirms that disrupting this bond rescues channel activity [220] and further supports the idea that structural rearrangements in the outer TM3/TM4 interface are crucial in mediating Orai1 pore opening.

### The P245 kink

In addition to the previously identified critical sites along the TM3/TM4 interface, a key residue is P245, located in the middle of TM4. This residue introduces a slight bend in the helix, effectively dividing it into TM4a and TM4b (Figure 3, P245–kink; Table 2). Notably, a mutation at this site (P245L) has been linked to the Stormorken-like syndrome, a condition associated with the constitutively active Orai1 channel, Orai1 P245L [92]. Substituting the conserved proline with any other canonical amino acid results in increased intracellular Ca^2+^ levels and nuclear translocation of NFAT in a STIM1-independent manner. This underscores the significant role of P245 in stabilizing channel closure and supports the hypothesis, based on structural analysis [149,150], that TM4 straightening underlies the observed channel activation [213]. Although straightening was proposed for the entire TM4/CT region, helicity analysis revealed distinct effects of ANSGA substitution on the TM4 proline bend and the nexus. These findings suggest that P245L-induced activation operated through a mechanism distinct from ANSGA mutation-triggered activation. In light of the critical role of P245, the proposed widening at the TM3/TM4 interface may represent a mechanism linked to the straightening of P245 within the central region of TM4, a conformational change required to trigger Orai channel pore opening.

Collectively, these studies highlight the critical role of the channel periphery in pore opening, in particular the interaction patterns and local distances in the TM3-TM4 contact interface, which are dynamically modulated by STIM1 association. However, further investigations are required to understand whether, in addition to a widening of non-pore-lining TM interfaces, other structural rearrangements, such as TM tilting, are induced by STIM1-coupling. Additionally, TM3/TM4b interface includes a prominent hydrophobic cluster of interdigitating bulky residues (L174, V181, I182, F250, F253, F257), which are thought to play a key role in maintaining the closed state of the channel. Nevertheless, the functional significance of this cluster in channel gating and structural stability requires further investigations.

## Structural rearrangements at the inner interface (TM2/3 and TM1)

Global conformational changes transferring the activation signal from the C-terminus to the pore of Orai1 involve not only signal transmission along the outer, but also the inner interface formed by TM2/3 and TM1. Atomic packing and hydrophobicity analysis, based on the crystal structure of dOrai1 (PDB: 4HKR) [149], was carried out to examine how individual TM sites contribute to channel gating [64]. The results revealed that TM2 and TM3 form a tightly packed helix pair with a more uniform packing density across their interfaces compared to TM1 or TM4. This structural arrangement suggests that TM2 and TM3 function as a rigid unit, transmitting the activation signal from the channel periphery (TM4) to the pore (TM1) via their shared interfaces, thereby facilitating pore opening. In the previous chapters, we highlighted potential contact sites and dynamics at the outer interface (TM4–TM3/2). We now turn to the inner interface (TM2/3–TM1) to outline key contact sites, interactions, and dynamic features involved in channel activation.

**Figure 5.**
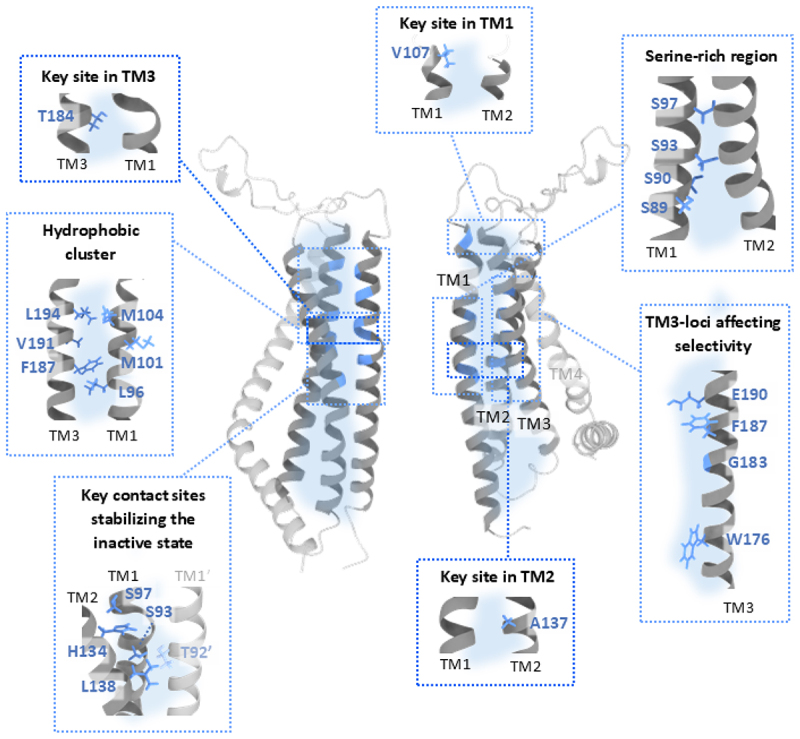
Critical sites within the inner interface of the Orai1 channel.

Functional studies have implicated residues in TM3 at the inner interface in shaping the ion selectivity of the Orai1 channel, particularly through E190 [64,159–163](Figure 5, TM3-loci affecting selectivity; Table 3). Although E190 does not directly line the pore, its position at the inner interface allows it to exert an allosteric influence on pore architecture and ion permeation. The E190Q substitution reduces Ca^2+^ selectivity and increases the pore diameter from 3.8 Å to 7.0 Å [225], yet cross-linking experiments show that E190 side chains are spatially distant and do not form a Ca^2+^ binding site. The E190A mutation does not affect Ca^2+^ influx, supporting an indirect role in ion permeation through allosteric modulation of the pore [163]. MD simulations performed on dOrai highlight the conserved TM3 residue E262 (analogous to E190 in Orai1) as critical for Ca^2+^ selectivity via regulation of pore hydration. K270 adopts a flexible orientation with the side chain pointing both into the extracellular domain and into the channel interior, leaving CAR unaffected. In contrast, the E262Q mutation reduces water density at the inner interface near the extracellular side, shifting K270 (analogous to K198 in Orai1) toward the selectivity filter and weakening ion coordination. Brownian dynamics simulations confirmed that this altered configuration of K270 in the mutant channel disrupts the electrostatic environment near CAR, indirectly affecting Ca^2+^ selectivity via altered structural CAR preferences [226]. A potential physiological consequence of hydration at the back of the selectivity filter might be a possible role of E190 as pH sensor, thus, affecting the conductance of the channel. Furthermore, hydration of this area might reduce energetic costs of channel gating involving the rotation of TM1 around the hydrophobic cavity [226].

Cross-sectional view of the hexameric Orai1 channel displaying two opposing subunits. TM3, TM2, and TM1 forming the inner interface (blue-shaded region) are shown in dark gray, while the nonparticipating TM4 is depicted in light gray. Critical residues in the backbone within the inner interface are highlighted in blue. Insets show the side chains of these residues, grouped by their functional role or location: Key site in TM3: T184. Hydrophobic cluster: L96, M101, M104, F187, V191, and L194. Key contact sites stabilizing the inactive state: T92,’ S93, S97, H134, and L138. TM1′ of the neighboring subunit is shown in light gray, with T92’ highlighted in light blue. Key site in TM1: V107. Key site in TM2: A137. Serine-rich region: S89, S90, S93, and S97. TM3-loci affecting selectivity: W176, G183, F187, and E190. Some insets show the TM domains rotated by specific angles to enhance visualization of the highlighted residues.

**Table 3.** Crucial residues along the Orai1 inner interface. Summary of critical residues located at the inner interface, their associated gain-of-function (GoF) and loss-of-function (LoF) mutations, relevance in disease, and currently known mechanisms versus unknown mechanisms and open questions.

Inter-face	Residues	GoF mutation	LoF mutation/reduced activity	Disease related	Known mechanism/notes	Unknown mechanism/open questions	Ref
Inner interface	E190	E190Q			Allosteric influence on pore architecture likely increases pore diameter and hydration	Mechanism causing non-selectivity (the direct or allosteric modulation on the pore) is not identified; contact sites are not mapped. (how these residues maintain the selectivity of Orai1)	[64,159–163,225]
	W176	W176C			Modulates Orai1 gating and selectivity		[64,207]
	G183	G183A					
	G183, F187	G183Azi/Bpa, F187Azi/Bpa			UAA insertion and/or photocrosslinking interfere with Ca^2+^ selectivity		[201]
	T184				T184 in TM3 may facilitate activation signal transmission after STIM1 binding		[206,208]
		T184M		Tubular Aggregate Myopathy	The methionine might introduce helix kink; facilitating activation signal transmission from TM4 to TM2		
	1) H134-S93/S97				H-bond is formed between H134 and S93/S97 stabilizing closed state		[151,202]
		H134A/C/S/T			Disrupt H-bond; induce constitutive activity		
	2) H134-S97				Steric hindrance by H134 impairs gating.		[64]
			H134F/Y/W		Inward-directed force at H134, hindering activation		
		H134A/S/C/T/V/M/E			Small substitutions at H134 create a steric void, promoting activation		
	A137	A137Bpa			Photocrosslinking-induced activation	Unknown direct functional relevant interaction partner	[200]
		A137V		Colorectal tumor	TM2 mutants enhance pore flexibility via stronger hydrophobic contacts	Spatial dynamics of the inner interface, particularly within this region, during pore opening remain incompletely understood	[202]
	L138	L138F		Myopathy			[203]
					Clockwise rotation of L138F, dilating the basic region within the pore		[227]
	L138-T92	L138F/Y	L138A/S/C/T		Bulky L138 substitutions activate the channel via steric pressure; L138-T92 inter-subunit contact		[205]
		T92L/M/F/Y/W	T92A/S/C/T				
	L96, M101, M104, F187, V191, L194				Hydrophobic cluster at the inner interface essential for proper STIM1-induced activation		[64]
	S89, S90, S93, S97				“Serine ridge” forming interaction with TM2/3 is essential for gating		
		S97C		Tubular Aggregate Myopathy	Disrupt polar contact with TM2/3 that stabilizes the closed state		[228]
	V107		V107M	Tubular Aggregate Myopathy	Key role in Orai1 gating and selectivity, probably due to the proximity to the selectivity filter	Unknown mechanism	[206,208]

Other studies have also identified F187, G183, and W176 in TM3 as key modulators of gating and selectivity [64,207] (Figure 5, TM3-loci affecting selectivity; Table 3). Mutations such as W176C and G183A lead to constitutive channel activation and altered Ca^2+^ -dependent block of monovalent ions. In line with these observations, Orai1 G183Azi/Bpa and Orai1 F187Azi/Bpa exhibited UV-induced activation; however, this was accompanied by pronounced nonselective currents. Notably, Orai1-G183Azi/Bpa also displayed constitutive activity associated with nonselective currents prior to UV light application [201]. These findings underscore further the regulatory role of TM3 in stabilizing the selectivity filter and maintaining the closed state of the channel, thereby fine-tuning Ca^2+^ influx and ion discrimination [207].

The Orai1 T184M mutation, located in TM3 and linked to TAM [206,208], displays a STIM1-dependent GoF phenotype without affecting Ca^2+^ selectivity (Figure 5, key site in TM3; Table 3). Interestingly, it is gated by STIM1-F394H, which does not activate wild-type Orai1. Functional gating via this STIM1-mutant is abolished by the Orai1 C-terminal mutations (L273D-L276D), known to disrupt STIM1 coupling. Bulla et al. [208] proposed that substituting threonine with methionine at position 184 may introduce a kink in the TM3 helix, potentially facilitating activation signal transmission from TM4 to TM2. T184M does not alter inhibition by acidic pH, suggesting preserved accessibility of pH-sensing residues. Although it may reduce solvent accessibility of C195, a Reactive-Oxygen-Species (ROS)-sensitive cysteine, MD simulations show this effect only at alkaline pH. Ca^2+^ imaging confirms normal H_2_O_2_ sensitivity under physiological conditions. Enhanced activity of Orai1 T184M in the presence of STIM1 has been associated with enhanced hydration along the cytosolic part of the pore, while MD simulations show unaltered side chain orientation of critical pore residing residues compared to the closed state [208].

Moreover, one of the most prominent sites within the inner interface, H134, is located in the middle of TM2. Its replacement by various amino acids (H134A/C/S/T) led to constitutive activity, exhibiting biophysical properties similar to those of the CRAC channel currents, except for fast Ca^2+^ -dependent inactivation (FCDI) [64,202]. As a result, this gating hotspot has been thoroughly investigated both functionally and structurally. In support of the gain of function behavior, MD simulations revealed an entirely wetted pore for dOrai1 H206A (analogous to Orai1 H134A) in contrast to wild-type dOrai [58,64]. Moreover, MD simulations on the Orai1 model exhibited a change in the pore conformation of Orai1 H134A versus wild-type Orai1 [202]. However, its precise mechanism remains a topic of debate, with two competing models proposed. Cysteine scanning along the pore revealed reduced crosslinking of V105C and V102C in Orai1 H134A, likely due to reduced TM1 flexibility, while crosslinking was more efficient in the hydrophobic segment (F99-S93), compatible with an increased flexibility in the GoF mutant. Furthermore, this analysis indicated a notable electrostatic interaction between H134 and either S93 or S97 in TM1. Based on these findings, they proposed that a hydrogen bond between H134 and these residues stabilizes the closed state of the channel (Figure 5, key contact sites stabilizing the inactive state; Table 3). In support of this hypothesis, disrupting this interaction, such as by mutating H134 to alanine (H134A), is suggested to induce gating by shifting the channel conformation to the open state. This model was further validated by the recently published high-resolution cryo-EM structure of dOrai H206A [151], which shows that substituting histidine with alanine disrupts the hydrogen bond with S93 and abolishes van der Waals interactions, destabilizing the closed state. However, Yeung et al. [64] proposed an alternative model challenging this hypothesis. They systematically substituted H134 with all amino acids and identified a correlation between current density and side-chain surface area, but not hydrophobicity. Larger side chains progressively reduced or entirely abolished channel activation (e.g. H134F/Y/W), suggesting that the size of the residue at this position plays a critical role. Based on these findings, they suggested that the bulky histidine (H134) functions as a steric brake, restricting channel opening by physically obstructing conformational changes. Replacing H134 with a smaller residue (e.g. H134A/S/C/T/V/M/E) removes this steric hindrance, allowing the channel to open (Table 3). This alternative model highlights the importance of steric effects at H134 in regulating Orai1 gating.

Residues in the central region of TM2 and close proximity to H134, such as A137 (Figure 5, key site in TM2; Table 3) and L138 (Figure 5, key sites contact stabilizing the inactive state; Table 3), are also critical for Orai1 pore opening. Their respective point mutations, Orai1 A137V, identified in a colorectal tumor [202], and Orai1-L138F, linked to myopathy [203], lead to constitutive current, with Orai1 A137V exhibiting a slightly nonselective current profile. Insights from MD simulations and cysteine scanning indicate that these mutations strengthen hydrophobic contacts within the pore, enhancing its structural flexibility. This, in turn, leads to a subtle expansion of the hydrophobic region within the pore, promoting the formation of a continuous water chain, which is believed to facilitate channel activation [202]. Similar findings were detected by another MD simulation performed by Zhang et al. [227], based on the crystal structure of dOrai, to examine the structural dynamics and hydration profiles of the L210F mutant (analogous to Orai1 L138F). Their analysis revealed two critical conformational changes: a clockwise rotation of F210, which dilates the basic region and K163 gate (Orai1 R91 analogous), and a counterclockwise rotation of F171 (Orai1 F99 analogous) in the hydrophobic gate, enhancing hydration. These combined motions are likely responsible for the constitutive activation of the L138F mutant channel and further highlight the role of TM2-TM1 hydrophobic interactions in pore gating [227].

An integrated study using MD simulations and functional assays explored further the mechanism of the Orai1 L138F mutation. Functionally, substituting L138 with various amino acids revealed that bulky side chains (L138F/Y) promote GoF activity, while smaller residues (e.g. L138A/S/C/T) result in LoF. Further analysis identified T92 in TM1 of the adjacent subunit (TM1’) as a likely interaction partner (Figure 5, key contact sites stabilizing the inactive state; Table 3), showing similar size-dependent effects on channel activity: small substitutions (T92A/S/C/T) did not show any activity, whereas larger residues (T92L/M/F/Y/W) induced strong constitutive activation. Atomic packing analysis indicated that L138F interacts primarily with TM1, and the GoF effect likely results from a steric clash between the introduced phenyl ring and a neighboring TM1 residue. Generally, they proposed that H134 and L138 show a coordinated role and act as pivot points of a structural brake that regulates the stability of the TM1 pore helix. Their distinct spatial arrangements, H134 interacting intra-subunit with S97 while L138 inter-subunit with T92, suggest they apply complementary mechanical forces on TM1. Specifically, bulky substitutions at L138 introduce steric pressure, while small substitutions at H134 create a steric void, both promoting channel activation. Conversely, inward-directed force at H134 or pulling at L138 disrupts this balance, resulting in LoF [205] (Figure 5, key contact sites stabilizing the inactive state; Table 3). Notably, both Orai1 T92W and Orai1 L138W mutants exhibit the typical biophysical hallmarks of the CRAC channel, even in the absence of STIM1. These represent the first reported Orai1 variants displaying rapid FCDI without STIM1, challenging the prevailing notion that STIM1 is essential for inactivation. This observation strongly suggests that the Ca^2+^-sensing machinery may be intrinsic to the Orai1 protein itself. Furthermore, Orai1 T92W exhibits enhanced Ca^2+^ sensitivity, which can be restored to normal levels in the presence of STIM1, highlighting the modulatory role of STIM1 in channel gating [205]. Similar to this finding, our recent study revealed that site-specific incorporation of Bpa at residue A137 (Figure 5, key site in TM2; Table 3) yields minimal basal channel activity prior to UV exposure but induces a robust Ca^2+^ influx post-UV irradiation. Notably, this UV-triggered activation shows the characteristic biophysical hallmarks of CRAC channels, with a particularly prominent FCDI profile, both in the presence and absence of STIM1 [200]. Collectively, these results emphasize the need for deeper exploration of the conformational dynamics of Orai1 A137Bpa and the UV-induced interactions, which may illuminate key mechanisms underlying Orai1 channel activation and Ca^2+^ permeation.

Notably, hydrophobicity at the inner interface plays a crucial role in relaying the gating signal to the central pore. A hydrophobic cluster near the extracellular side, including residues L96, M101, M104, F187, V191, and L194, creates a stack between TM1 and the TM2/3 ring [64] (Figure 5, hydrophobic cluster; Table 3). Mutations that disrupt these interactions eliminate communication between TM1 and other helices, impairing STIM1-mediated gating. Closer to the cytosolic side, a “serine ridge” formed by S89, S90, S93, and S97 lines the TM1 helix (Figure 5, serine-rich region; Table 3), interspersed with hydrophobic residues such as L96. This positioning creates alternating polar and nonpolar bands that align with complementary patterns on the TM2/3 ring, supporting flexible interactions essential for gating. The S97C mutation, linked to TAM [228], likely disrupts these polar contacts, destabilizing the closed state and promoting channel activation [64].

In extension, the TAM-associated V107M mutation near the selectivity filter [206,208], induces STIM1-independent channel activation and reduces Ca^2+^ selectivity, thereby increasing permeability to other cations (Figure 5, key site in TM1; Table 3). This mutation also diminishes inhibition by acidic pH, indicating altered gating sensitivity. In the presence of STIM1, Orai1 V107M channels exhibit larger STIM1-gated currents compared to wild-type Orai1. Despite their constitutive activity, these channels retain FCDI, suggesting partial preservation of regulatory mechanisms. Structural analyses using MD simulations revealed no major changes in hydration or orientation of pore-lining residues in Orai1 V107M relative to the wild-type channel. These findings suggest that V107 plays a central role in the pore-opening mechanism of Orai1; however, the precise molecular details underlying its contribution to channel gating remain to be fully elucidated. Notably, the Orai1 blocker GSK-7975A effectively inhibits V107M-mediated constitutive activity, highlighting its potential therapeutic relevance [208].

Overall, the hydrophobic character of the inner interface has been shown to play a main role in mediating Orai1 pore opening, alongside the contribution of specific residues that are essential for maintaining both ion selectivity and channel gating. Despite these insights, the spatial dynamics of the inner interface during pore opening remain incompletely understood. In particular, it is unclear whether the distances between TM helices, specifically the interface between TM1/TM2 and/or TM1/TM3, undergo expansion or constriction upon channel activation. Furthermore, while previous studies have proposed rotational movements within the pore during opening [64,166,192,208,210] (see next chapter), it remains to be determined whether additional rotational rearrangements occur within TM2 or TM3 that may contribute to the gating mechanism.

## Structural rearrangements of pore-lining residues

Finally, after the gating signal is transmitted from the periphery to the center of the channel complex, the central pore undergoes a series of conformational changes that allow Ca^2+^ influx.

**Figure 6.**
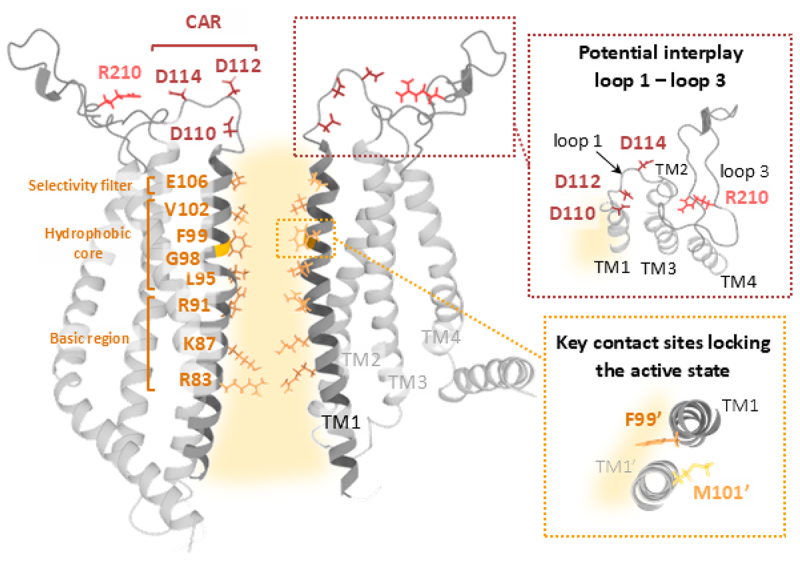
Critical sites within the pore and extracellular loops of the Orai1 channel.

Particularly important for maintaining Ca^2+^ selectivity of Orai1 is a glutamate ring composed of six E106 residues, which forms the selectivity filter at the pore entrance. Based on the crystal structure of the closed dOrai1, E178 (corresponding to Orai1 E106) (Figure 6; Table 4) defines the narrowest region of the pore (~6 Å) and generates a strongly negative electrostatic potential with high cation and particularly Ca^2+^ binding affinity, thereby defining cation selectivity in combination with steric effects [29,159–162]. Crosslinking experiments with the E106C mutant have provided evidence for the close proximity of E106 side chains in wild-type Orai1 [152,163]. Substitution of E106 with alanine, cysteine, or glutamine (E106A/C/Q) results in LoF phenotypes, effectively abolishing Ca^2+^ conductance through the Orai1 channel [46,159,160,162,225,229]. Moreover, the introduction of a single E106Q mutation into a concatenated Orai1 hexamer is sufficient to prevent Ca^2+^ influx [230], underscoring the critical role of E106 in channel function. Electrophysiological studies further confirmed the importance of the E106 carboxyl groups in forming Ca^2+^ binding sites. Replacing E106 with aspartic acid (E106D), which differs from glutamic acid by only one carbon atom in the side chain, significantly impairs Ca^2+^ selectivity and leads to nonselective permeation of monovalent cations such as cesium ions (Cs^+^) [159,225]. This shift toward nonselective permeation, despite minimal changes in electrostatic properties, highlights the need for precise spatial positioning of E106. This requirement is further supported by structural data from closed and open (H206A) states of dOrai1 [151]. In the closed state, the E106 side chains point downward, whereas the open cryo-EM structure of dOrai1 H206A reveals an alternating upward and downward orientation. This reorientation likely affects Ca^2+^ coordination and contributes to the formation of enthalpically-driven free energy barriers for ion passage, which are essential for maintaining Ca^2+^ selectivity [231].

Cross-sectional view of the hexameric Orai1 channel displaying two opposing subunits. TM1, which forms the pore (yellow-shaded region), is shown in dark gray, while the nonparticipating TM2, TM3, and TM4 are depicted in light gray. Critical pore-lining residues are highlighted in brown (Selectivity filter: E106, hydrophobic core: V102, F99, G98, L95, basic region: R91, K87, R83). Key residues in the extracellular loops are highlighted in dark red (CAR region in loop 1: D110, D112, D114) and light red (Loop 3: R210). Insets show side chains of selected residues within the pore (Key contact sites locking the active state: F99 and M101) and within the extracellular loops (Potential interplay loop 1 – loop 3: D112 and R210). The TM domains are shown rotated by specific angles to enhance visualization of the highlighted residues.

**Table 4.** Crucial pore lining residues. Summary of critical residues along the pore, their associated gain-of-function (GoF) and loss-of-function (LoF) mutations, relevance in disease, and currently known mechanisms versus unknown mechanisms and open questions.

Interface/region	Residues	GoF mutation	LoF mutation/reduced activity	Disease related	Known mechanism/notes	Unknown mechanism/open questions	Ref
Pore-lining residues	E106				Selectivity filter forms the Ca^2+^ binding site and the narrowest region in the pore		[29,159–162]
			E106A/C/Q		Substituting the E106 charge disrupts Ca^2+^ conductance		[46,159,160,162,225,229]
		E106D			Altering spatial positioning, despite preserved negative charge, disrupts Ca^2+^ selectivity		[159,225]
	V102, F99, L95				Hydrophobic gate restricts water and ion permeation	Is the rotation proposed within this region of the pore physiologically relevant?	[164,166]
		F99C/S/T/G/M/Y, V102A/C			Reduce hydrophobicity; induce constitutive activity		[162,163,166]
			F99L/V/I		Increase hydrophobicity; block activation		
	G98				“Gating hinge,” switching the channel between the open and closed states		[165]
			G98A		Reduces backbone flexibility of the pore; abolishes STIM1-induced activation		
		G98D/P				Mechanism not fully known	
		G98S		Tubular Aggregate Myopathy			[203,208]
			G98R	Immunodeficiency	Complete loss of Orai1 expression		[234]
	F99, M101, F187				M101 switches interaction from F187 (intra) to F99 (inter) upon channel opening		[204]
	R91, K87, R83				Inner channel gate, basic region of pore undergoes a clear widening, critical for facilitating Ca^2+^ influx	Different mechanisms were proposed: anion plug, rotation of R91 toward S90, and hydration effects; mechanisms remain unconfirmed	[148,150,151]
			R91W	severe combined immunodeficiency	Hydrophobic substitution impairs channel gating		[28,236]
Loop1/3	D110, D112, D114				Ca^2+^ -accumulating region, attract Ca^2+^; transient binding sites	Structural details of these regions remain unresolved	[152]
	D112, R210		D112C R210C		Disulfide crosslinking reduces Ca^2+^ permeation		

Directly beneath the selectivity filter is the hydrophobic central pore region, which forms a tightly packed bundle in the closed state of the Orai1 channel. This region is stabilized by three rings of hydrophobic residues (L95, F99, and V102) that restrict water and ion permeation [164,166] (Figure 6; Table 3). Pore hydration within the hydrophobic gate appears to be closely linked to Ca^2+^ permeation. Mutations that reduce hydrophobicity at F99 (e.g. F99C/S/T/G/M/Y) result in constitutive channel activation, whereas substitutions that increase hydrophobicity (e.g. F99L/V/I) lead to channel inactivity, even in the presence of STIM1 [162,163,166]. Similarly, mutations at V102 that lower hydrophobicity (V102A/C) not only induce constitutive activity but also compromise ion selectivity, allowing substantial permeation of sodium (Na^+^) and other monovalent cations [166]. MD simulations on dOrai V174A (corresponding to Orai1 V102A) compared to wild-type conditions reveal minimal structural perturbations, but a decrease of the free-energy barrier from 11 kcal/mol under wild-type conditions to 7 kcal/mol for the mutant. Water molecules are more aligned in the wild-type than the V174A mutant channel [232], which is in accord with functional properties. Notably, STIM1 binding restores Ca^2+^ selectivity in these mutants, suggesting a reorientation of E106 side chains via STIM1 [153,164]. V102, located one helical turn below E106, likely plays a role in maintaining the correct positioning of E106 under resting conditions [164,166,233].

Electrophysiological investigations using water-soluble thiol reagents (e.g. cadmium ions (Cd^2+^)) in the background of the GoF-mutations V102A and H134A revealed a state-dependent accessibility of cysteines integrated at the pore-lining residues G98 and F99. While G98C was accessible in the STIM1-bound state, F99C was accessible in the STIM1-unbound state [64]. These results indicate a STIM1-induced rotation of TM1 around the hydrophobic regions. In support, MD simulations of wild-type dOrai and mutants V174A, F171Y, and F171V (corresponding to V102A, F99Y, and F99V in Orai1), revealed a pronounced counterclockwise shift of ~20° in the pore-forming region for V174A and F171Y. This counterclockwise shift was not observed in wild-type or in the LoF mutant F171V [64,166]. In accord with these findings, MD simulations of the GoF mutants H206S/C in dOrai [64] (equivalent to H134S/C in Orai1) show substantial counterclockwise rotations of the pore helix, 30 ± 1° and 34 ± 2°, respectively, compared to wild-type dOrai (17 ± 1°) and V174A dOrai (22 ± 2°; V102A in hOrai1) [64,166]. Interestingly, Frischauf et al. [202] reported no significant rotation of the pore helix in their MD simulations, while Tiffner et al. [58] described the rotation of F99. The reason for the different observations could lie in the different simulation times, but could depend on other varying simulation conditions and/or force field parameters. Targeted MD studies are in good agreement with the observation of TM1 rotation around the hydrophobic region [209](Table 4).

The flexibility required for helical rotation of TM1 is conferred by the hinge residue G98 (Figure 6; Table 4). Due to its small side chain, glycine allows substantially augmented backbone flexibility, thereby facilitating rotational mobility. Zhang et al. [165] proposed that G98 functions as “gating hinge,” switching the channel between the open and closed states. This has been demonstrated through conventional mutagenesis studies. Substitution of G98 with alanine (G98A), which reduces backbone flexibility, abolishes STIM1-induced channel activation. In contrast, mutations introducing charged or larger side chains (G98D/P) result in constitutive, nonselective channel activity, likely due to pore widening and disruption of the α-helical structure [165]. Interestingly, the Orai1 G98S mutation causes constitutive Ca^2+^ influx and is associated with TAM [203,208]. In contrast, the G98R mutation leads to complete loss of Orai1 expression, abolishing SOCE and resulting in immunodeficiency [234].

Additionally, MD simulations revealed increased inter-subunit contact frequency between F99 and M101 (Figure 6, key contact sites locking the active state; Table 4) in Orai1-H134A/S. This indicates a stabilizing sulfur-aromatic interaction between these residues that supports the rotated open conformation of TM1 [204]. In contrast, simulations of the LoF mutant M173L (M101L in Orai1) in dOrai showed a reduced rotation of F171 (F99 in Orai1) to only 10°, compared to 17° in wild-type dOrai. This diminished rotation leads to reduced pore hydration and de-wetting of the hydrophobic core, as M173L stabilizes F171 in a pore-facing orientation, contributing to the LoF phenotype. In the closed state, M101 is oriented toward TM3, enabling interaction with F187 and thereby supporting the closed conformation of the pore [204].

In comparison to the closed state, the open dOrai structures exhibit a pronounced widening of the basic region (R91, K87, R83) in the Orai1 pore (Figure 6; Table 4), which is believed to be critical for facilitating Ca^2+^ influx [148,150,151]. In addition, the cryo-EM dOrai H206A mutant revealed that the side chains of residues within this region do not exhibit significant rotational changes compared to the closed conformation [151]. However, their local electron density is considerably less defined than that of other pore-lining residues, indicating increased conformational flexibility. Notably, the basic region is the most rigid segment of the Orai1 pore, beginning with residue R91 [202]. Crosslinking experiments have revealed distinct side chain orientations of R91 in the closed and open (H134A) states of the channel. In the closed conformation, R91C residues are oriented inward toward the pore axis, enabling crosslinking. In contrast, this crosslinking capability is lost in the open H134A mutant, indicating a conformational shift [202]. Consistent with these findings, MD simulations show that in the wild-type channel, three out of six R91 side chains face into the pore, whereas in the constitutively open H134A mutant, all R91 side chains are rotated outward toward the pore circumference, forming hydrogen bonds with neighboring S90 residues. A similar outward reorientation of R91 is observed when a Ca^2+^ ion is pulled through the wild-type channel, mimicking the conformational changes seen in the H134A mutant. Furthermore, simulations of Orai1 R91G reveal enhanced pore widening, even greater than that observed in H134A alone. Interestingly, the R91G mutation by itself does not induce constitutive activity, suggesting that pore dilation alone is insufficient for channel activation and ion permeation [202]. Instead, local water accessibility around the basic region of the pore appears to be a critical determinant of efficient Ca^2+^ permeation [235]. In support, the wild-type channel exhibits minimal water content, R91G shows a modest increase, and H134A permits a continuous double chain of water molecules with water dipoles aligned along the pore axis. Notably, the R91W mutation in Orai1 is directly associated with severe combined immunodeficiency (SCID) and represents a profound LoF variant [28,236]. Despite normal expression, localization, and co-clustering with STIM1 following store depletion, R91W fails to generate CRAC channel currents in HEK 293 cells, indicating a critical defect in channel gating or ion permeation. Functional studies revealed that hydrophobic substitutions at position 91, such as R91W, severely disrupt channel activity, whereas hydrophilic residues preserve function regardless of charge [28,236]. This underscores the importance of hydrophobicity at this site in modulating channel behavior.

Another study suggests that Ca^2+^ influx through the Orai channel is influenced not only by the structural features of the channel pore, but also by the surrounding ionic environment. In particular, Dong et al. [237] conducted MD simulations to understand ion transport in the dOrai V174A mutant, which is analogous to the V102A mutation in Orai1. Their simulations compared ion behavior under two different TM voltage biases: −500 mV and +500 mV, which left the overall channel structure unaffected, but introduced subtle conformational changes in the pore. Notably, under the −500 mV bias, spontaneous Na^+^ influx and concomitant Cl^−^ efflux were observed. Dong et al. [237] proposed an anion-assisted cation permeation mechanism, by which Cl^−^ ions cluster transiently with Na^+^ in the channel pore, facilitating their passage. This directional movement was absent under the +500 mV bias. These findings conjecture an active role of Cl^−^ counterions in facilitating cation permeation, particularly by modulating local electrostatics along the polybasic region for the Na^+^ ion in the lower pore. In line, several studies have proposed that cation – anion interactions within the basic region of the pore contribute to Ca^2+^ influx during the open state of the Orai1 channel [148,151,232]. Conversely, other reports suggest that larger anions (e.g. phosphates, pyrophosphates [149,238]) may be attracted to positively charged residues, collectively acting as a plug that stabilizes the closed conformation of the channel [149]. Computationally reported increase in MD equilibration time for RMSD depending on counterions and protonation states of titratable residues implies a crucial role of local pH in stabilizing the overall channel complex [232,238].

These contrasting mechanisms underscore the complex interplay between ionic dynamics and Orai1 channel gating behavior, which still needs further investigation. Another study that combined MD simulations with functional mutagenesis showed that neither the anion plug nor the proposed rotation of R91 is essential for Orai1 pore opening. Instead, the MD simulations revealed that positively charged residues within the basic region promote pore hydration, which is crucial for destabilizing the outer hydrophobic gate formed by F99 and V102. When these basic residues were neutralized, the simulations showed locally reduced water occupancy and stabilization of the closed conformation. Functional assays confirmed that these mutations abolished channel activation, both in the presence and absence of STIM1. Interestingly, introducing the V102A mutation restored channel activity, demonstrating a long-range coupling between the inner pore and the outer gate [235].

In summary, extensive studies have been conducted to elucidate the architecture of the Orai1 pore and its gating mechanisms. The role of the selectivity filter in maintaining ion selectivity is well established. Additionally, experimental, structural and computational analyses have revealed rotational movements within the hydrophobic region, particularly involving residue F99, that appear to facilitate pore opening. However, whether similar rotational dynamics occur within the basic region remains experimentally unverified. Based on available structural data, a pronounced widening of the basic region is observed in open channel conformations, suggesting its importance in enabling Ca^2+^ influx. Nevertheless, it remains a subject of ongoing debate whether this observed widening or rotation within the pore reflects a physiologically relevant dynamic process directly triggered by STIM1 during channel activation.

## Structural rearrangements of extracellular regions

The Orai1 pore features a wide outer vestibule lined with three aspartate residues (D110/D112/D114) (Figure 6; Table 4), which are located significantly near the selectivity filter (E106) [159–162,239]. These acidic residues are essential for recruiting Ca^2+^ ions near the pore entrance and enhancing Ca^2+^ permeation, thereby terming this region as the CAR [152]. Functional studies, supported by MD simulations, have demonstrated that mutating any of these residues reduced Ca^2+^ permeation [152]. MD simulations further reveal that transient Ca^2+^ binding occurs at Asp110 and Asp112 within the extracellular loop1. When exposed to 10 mM extracellular Ca^2+^, this region experiences a local surge in Ca^2+^ concentration, reaching 2.5 M within a 2 nm^3^ volume. The simulations also indicate the presence of a local energy barrier separating the CAR from the selectivity filter, which serves to prevent uncontrolled Ca^2+^ flow into the pore. Recent cryo-EM analysis of dOrai1 H206A mutant showed that the CAR forms part of a turret-like structure extending approximately 20 Å above the selectivity filter [151]. This structural feature further highlights the importance of the CAR in regulating Ca^2+^ entry and ensuring efficient ion selectivity and gating.

Interestingly, MD simulations identified loop3 as the most flexible region in Orai1 and revealed that D112 in loop1 transiently interacts with R210 in loop3 through electrostatic coupling (Figure 6, potential interplay loop1–loop3; Table 4). Distance measurements over a 100 ns simulation showed dynamic yet recurring interactions between loop1-loop3, with both intra- and intermonomer contacts forming intermittently. These simulations accurately predicted disulfide crosslinking between D112C and R210C, confirming a preferred geometric arrangement that is crucial for Orai1 channel function [152]. This finding was further validated by functional studies, which demonstrated that crosslinking of D112C in loop1 with R210C in loop3 reduces Ca^2+^ permeation in a Ca^2+^-dependent and steric manner. Conversely, disrupting this interaction, either by breaking the crosslink or introducing mutations that prevent loop1 and loop3 from interacting, enhances Ca^2+^ permeation [152]. These results highlight the importance of the loop1-loop3 interaction in fine-tuning Orai1 channel gating and ion permeation.

Taken together, the extracellular regions of the Orai channel do not only serve as connectors between TM domains but also contribute significantly to ion selectivity and Ca^2+^ entry into the pore. Despite their functional importance, the structural details of these regions remain unresolved and are still awaited. Elucidating their architecture and functional dynamics could provide critical insights into their precise role in Orai channel gating. It is plausible that dynamic variations and potential conformational changes within these extracellular segments are necessary to initiate and facilitate Ca^2+^ influx and pore opening.

## Summary

CRAC channels are complexes formed by the ER-resident protein STIM and the PM channel Orai [3,5,14,27]. Physiological CRAC channel complexes consist of assemblies of the various available isoforms [26,30]. While significant progress has been made in understanding the activation mechanisms of CRAC channels [61,154,200,201,240], particularly the interplay between STIM1 and Orai1 [27,54,55,57,61,141], a series of details within this complex machinery still remain unresolved. It is well established that Orai1 is activated by STIM1 upon depletion of ER Ca^2+^ stores [19,20]. STIM1 couples to the C-terminus of Orai1, which serves as the primary coupling site [54–56,171,179,180]. This interaction triggers interdependent motions of the TM domains, ultimately leading to the opening of the Orai1 pore. The global conformational change required for pore opening involves a wave of coordinated TM domain motions [58,64,151,166,201,202,240]. These include a widening of the TM3/TM4 interface [201], dilation of the basic region [148–151], and rotation of the hydrophobic cavity within the pore [166] as consistently suggested by functional experiments, structural studies and MD simulations. The channel’s high Ca^2+^ selectivity is maintained by its selectivity filter [149,225], which is further regulated by the extracellular CAR [152,225] and key residues in TM3 [64,159–163,207,226]. Despite this growing body of knowledge, several critical questions remain unanswered (see below). Addressing these points will require extensive interdisciplinary efforts, further embracing molecular modeling, docking, and dynamics approaches, which will be essential to complement experimental findings and achieve an atomistic understanding of the CRAC channel activation cascade.

## Perspectives

Although the Orai1 C-terminus is well established as the primary coupling site for STIM1 [54–56,171,179,180], the atomic-level details of how the functionally critical N-terminus and loop2 region contribute to this interaction remain unresolved. Beyond the structural rearrangements observed in individual TM domains, the motions of the TM2/3 ring, which lies between the TM4 and TM1 rings, as well as the dynamics of the cytosolic regions, are still unknown. Notably, the minimal structural motions observed within the TM domains, as well as in the extra- and intracellular regions, remain incompletely resolved in the currently available structural data and require further experimental investigations. Further studies are needed to fully elucidate subtle yet critical rearrangements, such as narrowing or widening of specific interfaces, or rotational shifts within particular TM domains. These details are essential for a comprehensive understanding of the channel’s activation mechanism. Dynamic approaches using light-sensitive unnatural amino acids offer a promising avenue for resolving these gaps. However, the ultimate goal remains to obtain a high-resolution structure of STIM1 in complex with Orai1.

While significant progress has been made in understanding the interplay between STIM1 and Orai1 [54,61,153,155], the diverse activation mechanisms of CRAC channels [61,154,201,240,241], particularly those involving distinct stoichiometries [6,193–195,197,198,230,242,243] and isoforms [3,36,56,174,178,221,244–249], remain poorly understood. Given that the CRAC channel machinery operates at ER-PM junctions, future research must focus on elucidating the roles and binding sites of modulatory proteins in this context.

In addition to accessory proteins [14,65–69], other factors such as phosphorylation, post-translational modifications [70–81,179], and lipid interactions [69,82–84] likely fine-tune CRAC channel activity. These factors may function in a cell type-specific manner, enabling the precise regulation of CRAC channels across different tissues. Addressing these open questions will be critical for achieving a comprehensive understanding of CRAC channel regulation and function.

Our recent extensive application of UAA mutagenesis to the Orai channels demonstrates its power for advancing our understanding of Ca^2+^ ion channels [200,201]. By incorporating UAAs with tailored physicochemical characteristics, researchers can probe specific aspects of CRAC channel structure, function, and dynamics with unprecedented precision. This approach enables the study of critical regions, such as binding interfaces, gating, (in-)activation mechanisms, and conformational changes, at the amino acid [217,250–254], which can be expanded to selectively reactive UAAs, allowing to precisely map contact sites [217,255–257]. Atomistic molecular docking approaches as well as MD simulations are perfectly suited to further scrutinize and complement the structural rearrangements and binding interfaces put forward by experiment. These computational approaches will likely be able to rationalize the modulatory role of the Orai1 termini in CRAC association and dissociation. The use of UAAs opens new dimensions in structural biology, allowing visualization of ion channels alone or in complex with accessory proteins or ligands. Additionally, light-sensitive UAAs can be employed to achieve spatiotemporal control over CRAC channel activity, offering insights into their real-time behavior in living cells. Such advancements have not only deepened our knowledge of CRAC channel biology and biophysics but have also paved the way for the development of novel therapeutic strategies targeting these channels in diseases where Ca^2+^ signaling is dysregulated.

## Data Availability

No new data were created or analyzed in this study. Data sharing is not applicable to this article.
